# A Review on PolSAR Decompositions for Feature Extraction

**DOI:** 10.3390/jimaging10040075

**Published:** 2024-03-24

**Authors:** Konstantinos Karachristos, Georgia Koukiou, Vassilis Anastassopoulos

**Affiliations:** Electronics Laboratory (ELLAB), Physics Department, University of Patras, 26504 Rio, Greece; gkoukiou@upatras.gr (G.K.); vassilis@upatras.gr (V.A.)

**Keywords:** PolSAR, coherent decomposition, non-coherent decomposition, feature extraction, Pauli decomposition, Cameron CTD, Freeman–Durden decomposition, Yamaguchi decomposition, H/A/a decomposition, double scatterer model

## Abstract

Feature extraction plays a pivotal role in processing remote sensing datasets, especially in the realm of fully polarimetric data. This review investigates a variety of polarimetric decomposition techniques aimed at extracting comprehensive information from polarimetric imagery. These techniques are categorized as coherent and non-coherent methods, depending on their assumptions about the distribution of information among polarimetric cells. The review explores well-established and innovative approaches in polarimetric decomposition within both categories. It begins with a thorough examination of the foundational Pauli decomposition, a key algorithm in this field. Within the coherent category, the Cameron target decomposition is extensively explored, shedding light on its underlying principles. Transitioning to the non-coherent domain, the review investigates the Freeman–Durden decomposition and its extension, the Yamaguchi’s approach. Additionally, the widely recognized eigenvector–eigenvalue decomposition introduced by Cloude and Pottier is scrutinized. Furthermore, each method undergoes experimental testing on the benchmark dataset of the broader Vancouver area, offering a robust analysis of their efficacy. The primary objective of this review is to systematically present well-established polarimetric decomposition algorithms, elucidating the underlying mathematical foundations of each. The aim is to facilitate a profound understanding of these approaches, coupled with insights into potential combinations for diverse applications.

## 1. Introduction

The use of remote sensing techniques in conjunction with advanced technological tools provides ample opportunities to study entire ecosystems [1,2,3,4,5,6,7] through the collection of vast amounts of data. The stage of information processing holds paramount significance [8,9] across diverse applications leveraging satellite data. Ongoing research endeavors aim to delve into myriad methods for extracting information from a spectrum of remote sensing data sources. As we stand on the cusp of artificial intelligence dominance, the exploration of machine learning techniques [10,11] and, more prominently, deep learning approaches is gaining traction for extracting intricate polarimetric features [12,13]. This emphasis is particularly pronounced in the domain of Polarimetric Synthetic Aperture Radar (PolSAR) [14,15,16,17,18].

PolSAR, also known as Quad-Pol data, enhances the information about the polarization status of electromagnetic waves. More precisely, a PolSAR system functions similarly to a conventional radar system by transmitting and receiving microwave signals. However, it additionally incorporates the capability to capture the various polarization states of the echoes received. By using different polarizations, it is possible to discern unique and distinct features of targets. Some features can be observed in one polarization but not in another. Radar systems have two common basis polarizations, horizontal linear (*H*) and vertical linear (*V*), which allow them to transmit and receive radio waves with either horizontal or vertical polarization. However, in polarimetric SAR, it is possible to transmit or receive radio waves with both polarizations, allowing for a more detailed analysis of the backscattered signals. Therefore, full PolSAR data captures all the polarization information available in the backscattered wave (*HH*, *HV*, *VH*, *VV*), represented in a 2 × 2 matrix form, known as polarimetric scattering matrix S, that describes the relationship between the incident and scattered electric fields of the waves.

(1) $$S=\left[\begin{array}{cc} S_{HH} & S_{HV}\\ S_{VH} & S_{VV} \end{array}\right]$$

Each element of the matrix corresponds to a specific polarization component of the scattered wave, and by analyzing the matrix, plenty of information can be provided about the physical properties of the scattering target. Polarimetric target decompositions, a class of algorithms, have been developed to extract diverse features from PolSAR images, enhancing their utility in classification and target detection tasks by capturing multiple aspects. Many of these algorithms have been proposed in the literature [15,16,17,18,19,20,21,22,23,24,25,26,27,28,29,30,31,32,33]. These methods can be broadly categorized as either coherent or non-coherent decomposition techniques [18]. Each of these methods tackles the task of interpreting the information contained in PolSAR cells by starting with the underlying assumption about the target. Coherent decompositions, as outlined in various studies [16,17,18,19,20,21,22], are grounded on the deterministic premise that each resolution cell represents a single, predominant scattering mechanism. Consequently, these techniques endeavor to disentangle this primary scattering behavior and link it to an elementary scatterer characterized by a simple geometric structure. This endeavor entails representing the backscattering matrix *S*, typically measured in the HV basis as a coherent aggregation of basis matrices, with each aligning with an elementary scattering mechanism.

(2) $$S=\sum_{k=1}^{N}a_{k}S_{k}$$

The scattering matrix of an elementary scattering mechanism k corresponds to $S_{k}$ and $a_{k}$ represents the weight of each scattering mechanism.

Conversely, non-coherent decomposition methods [23,24,25,26,27,28,29,30,31,32] have been formulated with the premise that the target scatters across multiple adjacent PolSAR cells, influenced by the speckle noise inherent in SAR imaging. In this case, since each resolution cell lacks a dominant scattering mechanism, the extraction of information requires the utilization of second-order statistics. Non-coherent targets, which exhibit inherent stochastic behavior, can be identified using the concept of covariance and coherency matrix. To accomplish this goal, a four-element complex vector is employed, containing the same information as the polarimetric scattering matrix. From this vector, the covariance and coherency matrices are then derived, as defined below:

(3) $$S=\left[\begin{array}{cc} S_{HH} & S_{HV}\\ S_{VH} & S_{VV} \end{array}\right]\to \vec{k}=\frac{1}{2}Trace\left(S\left[\Psi \right]\right)=\left[k_{0},\;k_{1},\;k_{2},\;k_{3}\right]$$

where Ψ represents a comprehensive set of 2 × 2 complex basis matrices under the Hermitian inner product [17]. When employing the lexicographic basis, also known as the Borgeaud basis:

(4) $$\Psi_{l}=\left\{2\left[\begin{array}{cc} 1 & 0\\ 0 & 0 \end{array}\right],\;2\left[\begin{array}{cc} 0 & 1\\ 0 & 0 \end{array}\right],\;2\left[\begin{array}{cc} 0 & 0\\ 1 & 0 \end{array}\right],\;2\left[\begin{array}{cc} 0 & 0\\ 0 & 1 \end{array}\right]\right\}$$

The corresponding vector is formatted as follows:

(5) $$k_{l}={\left[S_{HH},\;S_{HV},\;S_{VH},\;S_{VV}\right]}^{T}$$

and the polarimetric covariance matrix ${\left[C\right]}_{4}$ is constructed by the outer product $<{\vec{k}}_{l}{\vec{k}}_{l}^{†}>$ of the scattering vector ${\vec{k}}_{l}$ with its conjugate transposed ${\vec{k}}_{l}^{†}$:

(6) $${\langle \left[C_{4}\right]\rangle }_{v}=\left[\begin{array}{cccc} \langle {\left|S_{HH}\right|}^{2}\rangle & \langle S_{HH}S_{HV}^{*}\rangle & \langle S_{HH}S_{VH}^{*}\rangle & \langle S_{HH}S_{VV}^{*}\rangle \\ \langle S_{HV}S_{HH}^{*}\rangle & \langle {\left|S_{HV}\right|}^{2}\rangle & \langle S_{HV}S_{VH}^{*}\rangle & \langle S_{HV}S_{VV}^{*}\rangle \\ \langle S_{VH}S_{HH}^{*}\rangle & \langle S_{VH}S_{HV}^{*}\rangle & \langle {\left|S_{VH}\right|}^{2}\rangle & \langle S_{VH}S_{VV}^{*}\rangle \\ \langle S_{VV}S_{HH}^{*}\rangle & \langle S_{VV}S_{HV}^{*}\rangle & \langle S_{VV}S_{VH}^{*}\rangle & \langle {\left|S_{VV}\right|}^{2}\rangle \end{array}\right]$$

where 〈 〉 represents a spatial ensemble averaging assuming homogeneity of the random scattering medium.

Otherwise, by using another widely referred basis, the Pauli basis:

(7) $$\Psi_{p}=\left\{\sqrt{2}\left[\begin{array}{cc} 1 & 0\\ 0 & 1 \end{array}\right],\;\sqrt{2}\left[\begin{array}{cc} 1 & 0\\ 0 & -1 \end{array}\right],\;\sqrt{2}\left[\begin{array}{cc} 0 & 1\\ 1 & 0 \end{array}\right],\;\sqrt{2}\left[\begin{array}{cc} 0 & -i\\ i & 0 \end{array}\right]\right\}$$

The vector’s form is:

(8) $$k_{p}=\frac{1}{\sqrt{2}}{\left[S_{HH}+S_{VV},\;S_{HH}-S_{VV},\;S_{HV}+S_{VH},\;i(S_{VH}-S_{HV})\right]}^{T}$$

In a similar way the polarimetric coherency matrix is evaluated as follows:

(9) $${\langle \left[T_{4}\right]\rangle }_{v}=<{\vec{k}}_{p}{\vec{k}}_{p}^{†}>$$

Without ensemble averaging, both matrices depict a deterministic scattering mechanism [30]. By definition, these matrices are Hermitian semi-definite matrices with identical real non-negative eigenvalues. However, their eigenvectors differ.

According to [17], non-coherent decompositions can be divided into three categories. The first one consists of the decompositions that are based on the dichotomy of the Kennaugh matrix [23], the second one includes the so-called model-based decompositions [20,21,22,23,24,25], and the third one encompasses the algorithms that utilize the eigenvector analysis [26,27,28].

The present study aims to conduct a thorough analysis of fundamental decomposition techniques while also acknowledging the significance of other methods and recognizing the innovation of new ones. The structure of the review is outlined as follows: in Section 3, coherent decomposition techniques, including Pauli [17,18] and Cameron [20,21,22], will be subjected to in-depth analysis. Section 4 will focus on non-coherent techniques, specifically the model-based three-component Freeman–Durden decomposition [24] and the Yamaguchi four-component approach [25]. The Entropy-based decomposition introduced by S. R. Cloude and E. Pottier [30], serving as a cornerstone of eigenvector-based decomposition methods, will be discussed in Section 5. Section 6 will analyze the newly established Double Scatterer model approach [33]. A comprehensive evaluation of these techniques will be presented, drawing on experiments conducted using the benchmark Fully Polarimetric dataset covering the broader area of Vancouver. Subsequently, in Section 7, conclusions will be drawn based on the findings.

## 2. Dataset and Preprocessing

**Figure 1:**
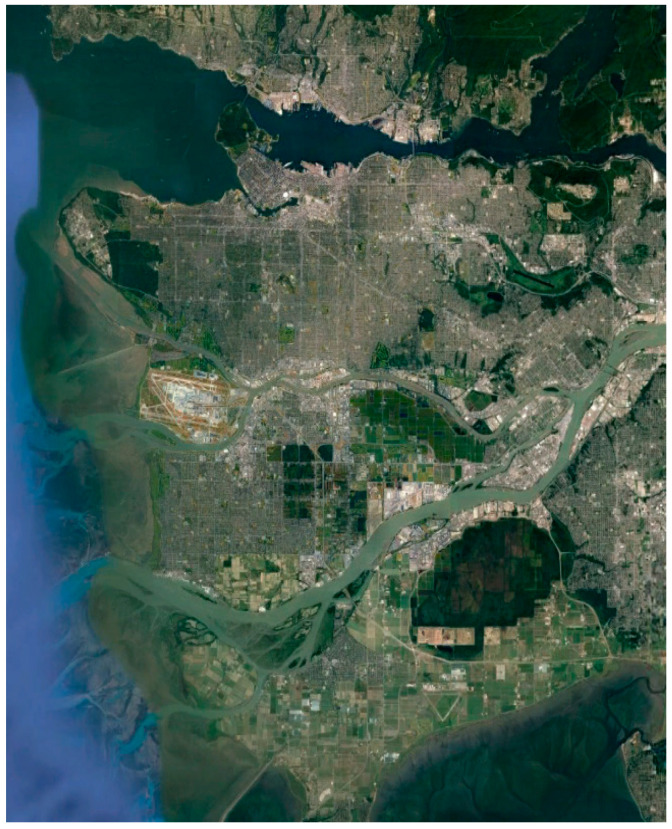
Broader area of Vancouver, by Google Earth.

The data product was obtained from the RADARSAT-2 satellite mission [34] in April 2008 and depicts the broader area of Vancouver (Figure 1), utilizing C-band. The image was captured using the Fine Quad-Pol Beam mode, offering fully polarimetric imaging with a specified resolution of 5.2 × 7.6 [Range × Azimuth] (m^2^) and swath widths of approximately 25 km.

**Figure 2:**
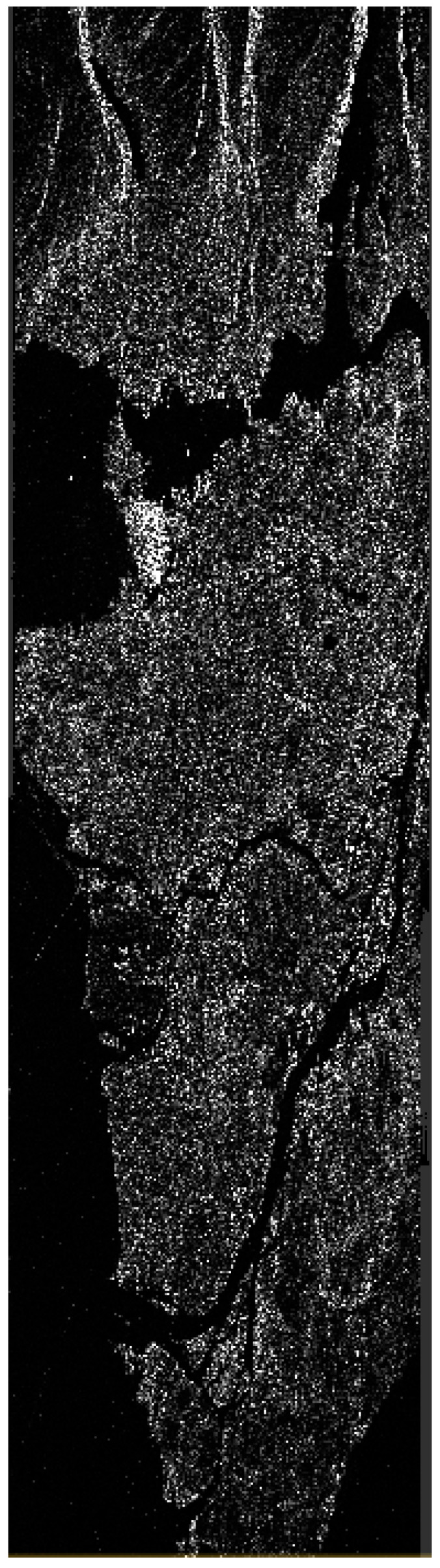
The broader area of Vancouver, as represented by the Intensity of the HV channel of PolSAR SLC data.

Among the available products, Single Look Complex (SLC) was chosen [35]. SLC products represent images in the slant range by azimuth imaging plane within the satellite data acquisition’s image plane. Each pixel in the image is denoted by a complex (*I* and *Q*) magnitude value, encompassing both amplitude and phase information. Each *I* and *Q* value is coded at 16 bits per pixel. Therefore, for each PolSAR image, a total of 12 components are available, including the *I* and *Q* components for each of the polarimetric acquisition (*HH*, *HV*, *VH*, *VV*), as well as the calculated intensity ($Intensity=\sqrt{I^{2}+Q^{2}}$) for each channel. During the processing of SLC image products, no interpolation into ground range coordinates occurs. Therefore, the range coordinate is expressed in radar slant range instead of ground range. In other words, the spacing between pixels and the resolution is determined along a slant path perpendicular to the sensor’s track (Figure 2). Pixel spacings are measured by the radar’s range sampling rate and pulse repetition frequency (PRF).

The selection of the specific dataset serves a dual purpose. Firstly, it is a freely available benchmark dataset widely used in research, offering valuable insights into the inherent characteristics of the specific polarimetric synthetic aperture radar (POLSAR) imagery. Secondly, and of paramount importance, the availability of the raw format provides us with flexibility and robustness. This allows us to process the data in various ways and conduct numerous experiments, affording the freedom to test our methods without being constrained by a standard processing path.

To effectively process PolSAR SLC data, it is essential to undergo radiometric calibration and terrain correction processes [36]. These processes were executed using the Sentinel Application Platform (version 8.0, SNAP) application, which serves as a common architecture for all Sentinel Toolboxes. Designed for Earth observation (EO) processing and analysis, the SNAP architecture is versatile and supports a wide array of sensors beyond the Sentinel series. The SNAP user tool is made available at no cost to the Earth Observation Community by ESA/ESRIN [37].

It is noteworthy to highlight the development of a specialized toolbox named PolSARpro. This toolbox, crafted by the European Space Agency (ESA), is dedicated to the processing and analysis of polarimetric synthetic aperture radar (PolSAR) data. POLSARPRO offers an extensive suite of tools encompassing calibration, visualization, and analysis functionalities for PolSAR data. This toolbox plays a pivotal role in facilitating the calibration and interpretation of polarimetric information derived from SAR observations.

**Figure 3:**
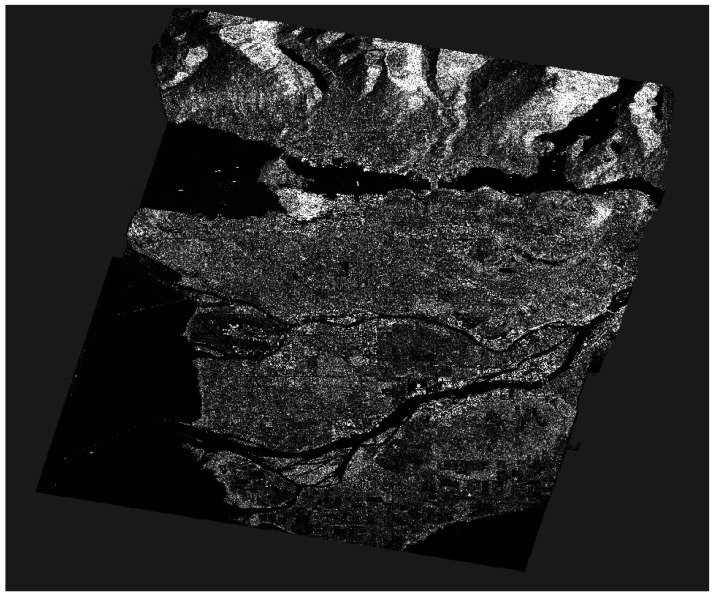
The HV channel intensity of PolSAR SLC data showcases the Vancouver region following preprocessing, which includes calibration and terrain correction.

Radiometric calibration plays a crucial role in standardizing the raw digital image data obtained from satellites into a consistent physical scale. This procedure depends on documented reflectance data collected from various objects present on the surface of the Earth. Since the image is acquired in the radar’s slant range, distortions associated with side-looking geometry may arise, and the pixels may lack geographical coordinates. To address this, the Range Doppler terrain correction operator available in SNAP was utilized. This method is employed to geocode SAR images and produce map-projected products (Figure 3). During this process, orbit state vector information from the metadata, radar timing annotations, parameters for converting slant to ground range, and data from the reference digital elevation model are integrated to determine precise geolocation information.

## 3. Coherent Target Decomposition

### 3.1. Pauli Target Decomposition

Pauli Decomposition expresses the polarimetric scattering matrix *S* into the complex sum of the Pauli spin matrices. Each one is multiplied by a complex coefficient and is associated with an elementary scattering mechanism:

(10) $$S=\left[\begin{array}{cc} S_{HH} & S_{HV}\\ S_{VH} & S_{VV} \end{array}\right]=\frac{a}{\sqrt{2}}S_{a}+\frac{b}{\sqrt{2}}S_{b}+\frac{c}{\sqrt{2}}S_{c}+\frac{d}{\sqrt{2}}S_{d}$$

where the basis set is presented as follows:

(11) $$S_{a}=\left[\begin{array}{cc} 1 & 0\\ 0 & 1 \end{array}\right]\;S_{b}=\left[\begin{array}{cc} 1 & 0\\ 0 & -1 \end{array}\right]\;S_{c}=\left[\begin{array}{cc} 0 & 1\\ 1 & 0 \end{array}\right]\;S_{d}=\left[\begin{array}{cc} 0 & -j\\ j & 0 \end{array}\right]$$

and the Pauli complex coefficients are provided next:

(12) $$a=\frac{{(S}_{HH}+S_{VV})}{\sqrt{2}}\;,\;b=\frac{{(S}_{HH}-S_{VV})}{\sqrt{2}},\;c=\frac{{(S}_{HV}+S_{VH})}{\sqrt{2}},\;d=j\frac{{(S}_{HV}-S_{VH})}{\sqrt{2}}$$

The Pauli complex coefficients reveal the strength of the four superimposed scattering mechanisms. These mechanisms are used to describe deterministic targets included in each PolSAR cell. The four elementary scattering mechanisms are:

(a) The single or odd bounce scattering mechanism, also referred to as the plate, sphere, or trihedral scattering mechanism, corresponds to the $S_{a}$ component.
(b) The diplane scattering mechanism, also referred to as dihedral scattering or, in general cases, as double or even bounce scattering from corners with a relative orientation of $0^{\circ }$, is presented by the $S_{b}$;
(c) and with a relative orientation of $45^{\circ }$, corresponds to the $S_{c}$ component.
(d) The Antisymmetric mechanisms are depicted via the $S_{d}$ component.

By analyzing the amplitudes of these four components, the scattering mechanisms in a PolSAR cell can be identified and evaluated.

In the context of the monostatic radar, employing a single antenna for both transmission and reception and relying on the reciprocity theorem, which states that $S_{HV}=S_{VH}$, the scattering matrix can be expressed as follows:

(13) $$S=\left[\begin{array}{cc} S_{HH} & S_{HV}\\ S_{HV} & S_{vv} \end{array}\right]=aS_{a}+bS_{b}+cS_{c}=a\left[\begin{array}{cc} 1 & 0\\ 0 & 1 \end{array}\right]+b\left[\begin{array}{cc} 1 & 0\\ 0 & -1 \end{array}\right]+c\left[\begin{array}{cc} 0 & 1\\ 1 & 0 \end{array}\right]$$

where

(14) $$a=\frac{{(S}_{HH}+S_{VV})}{\sqrt{2}}\;,\;b=\frac{{(S}_{HH}-S_{VV})}{\sqrt{2}},\;c=\sqrt{2}S_{HV}$$

The span of the reduced matrix *S* can be easily obtained as:

(15) $$SPAN={\left|S_{HH}\right|}^{2}+{\left|S_{VV}\right|}^{2}+2{\left|S_{HV}\right|}^{2}={\left|a\right|}^{2}+{\left|b\right|}^{2}+{\left|c\right|}^{2}$$

**Figure 4:**
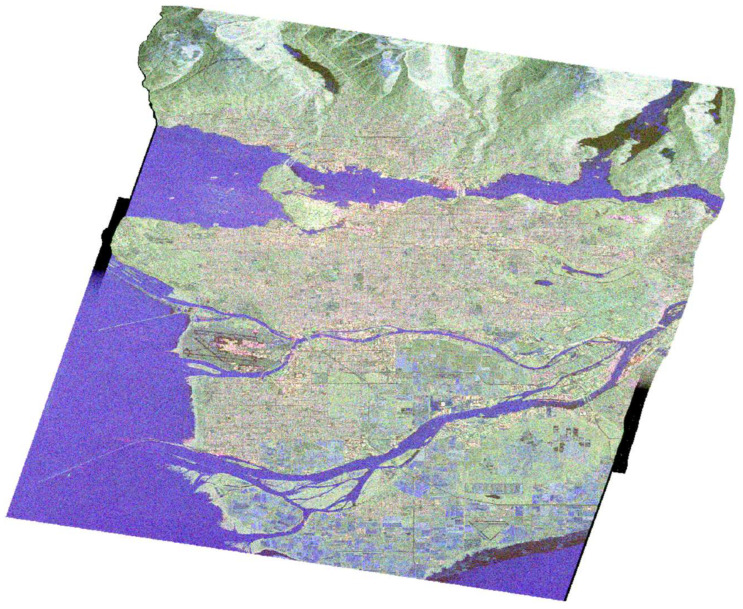
Color-coded PolSAR image of the broader area of Vancouver, based on the Pauli target decomposition: ${\left|a\right|}^{2}\to$ Red, ${\left|b\right|}^{2}\to$ Blue, ${\left|c\right|}^{2}\to$ Green.

The magnitude of the coefficients can provide valuable insights into each cell within a PolSAR image. By combining the magnitudes of the coefficients ${\left|a\right|}^{2},\;{\left|b\right|}^{2},\;\mathrm{and}\;{\left|c\right|}^{2}$, the polarimetric data of each cell can be visually represented in an RGB image. Each intensity corresponds to a physical scattering mechanism, as mentioned earlier, and is associated with a color image. The most frequently occurring match is the following:

(16) $${\left|a\right|}^{2}\to \mathrm{Red},\;{\left|b\right|}^{2}\to \mathrm{Blue},\;{\left|c\right|}^{2}\to \mathrm{Green}$$

and is depicted in Figure 4.

It is worth mentioning that a disadvantage of this decomposition is the lack of discrimination between the scattering mechanisms and the dependency on the orientation angles in the case of diplane/dihedral scattering mechanism. A comprehensive exploration of Pauli mechanisms is available in reference [38], providing detailed insights into the intricate interplay of these mechanisms. Additionally, the correlation between the degree of Linear Polarization and the Pauli color-coded image is thoroughly examined within the same reference, shedding light on their interconnected dynamics. At the application level, Pauli decomposition is widely used as a fundamental and computationally simple technique in various fields. It is often combined with other decomposition methods and machine learning algorithms for both classification and detection tasks. For example, in a study by O. Okwuashi et al. [39], Pauli decomposition was used in conjunction with the Gray Level Co-occurrence Matrix (GLCM) for texture feature extraction. The extracted information was utilized by a Deep Support Vector Machine model in land cover classification with 11 classes. The results were compared with other well-established approaches as concern the classifiers, and the accuracy of the specific procedure was highlighted. In [40], Pauli decomposition is employed in a land cover classification approach that is based on the idea of Super pixels and uses Convolutional Neural Networks. Furthermore, Fan. W. et al. [41] utilized Pauli decomposition in order to generate color-coded images and feed Deep Convolutional Neural Networks in a ship detection task, while in [42], Pauli’s, along with three other decomposition approaches, were employed in a ship detection study that combines algorithms aiming to improve the probability of target detection.

### 3.2. Cameron Target Decomposition

Cameron’s decomposition is based on the properties of reciprocity and symmetry. The decomposition involves two stages: the first stage is the decomposition of the scattering matrix into reciprocal and non-reciprocal components by means of the angle $\theta_{rec}$. The second stage decomposes the reciprocal term into symmetric and non-symmetric components using the angle $\tau_{sym}$. Cameron’s CTD analysis [20,21,22] utilizes the Huynen [23] hypothesis of the two fundamental properties of scatterers, reciprocity, and symmetry. A scatterer exhibits reciprocity when the off-diagonal elements of its backscattering matrix are equal in pairs. The principle of reciprocity extends to all monostatic SAR, as the same antenna is used for transmission and reception. Consequently, all scatterers are perceived as reciprocal when observed by monostatic SAR systems. Furthermore, a reciprocal scatterer is deemed symmetric if it displays an axis of symmetry perpendicular to the radar’s line of sight (LOS).

In accordance with Cameron’s algorithm, the backscattering matrix *S* is first mapped onto a basis set, where each matrix corresponds to an elementary scatterer. The basis set chosen is the Pauli matrices, Equation (7).

(17) $$S=\left[\begin{array}{cc} S_{HH} & S_{HV}\\ S_{VH} & S_{vv} \end{array}\right]=\frac{a}{\sqrt{2}}\left[\begin{array}{cc} 1 & 0\\ 0 & 1 \end{array}\right]+\frac{b}{\sqrt{2}}\left[\begin{array}{cc} 1 & 0\\ 0 & 1 \end{array}\right]+\frac{c}{\sqrt{2}}\left[\begin{array}{cc} 0 & 1\\ 1 & 0 \end{array}\right]+\frac{d}{\sqrt{2}}\left[\begin{array}{cc} 0 & -j\\ j & 0 \end{array}\right]$$

By converting the scattering matrix *S* into a vector format for computational ease and effectiveness, the ensuing expression is obtained:

(18) $$s=V\left(S\right)=\alpha {\hat{s}}_{a}+\beta {\hat{s}}_{b}+\gamma {\hat{s}}_{c}+\delta {\hat{s}}_{d}$$

The hat $\hat{s}$ of vector s symbolizes a unit vector ($\left|\hat{s}\right|=1,$ where |…| stands for vector magnitude).

Drawing from the reciprocity theorem, which stipulates that $S_{HV}=S_{VH}$, Cameron categorizes the target into either reciprocal or non-reciprocal, based on the projection angle $\theta_{rec}$ within the reciprocal subspace.

(19) $$\theta_{rec}=\cos^{-1}\left|\left|P_{rec}S\right|\right|,\;\;\;0\leq \theta_{rec}\leq \frac{\pi }{2}$$

where

(20) $$P_{rec}=\left(\begin{array}{cccc} 1 & 0 & 0 & 0\\ 0 & \frac{1}{2} & \frac{1}{2} & 0\\ 0 & \frac{1}{2} & \frac{1}{2} & 0\\ 0 & 0 & 0 & 1 \end{array}\right)$$

If $\theta_{rec}\leq 45^{\circ }$, the elementary scattering mechanism is considered as reciprocal, otherwise it is taken as non-reciprocal. The scattering matrix of a reciprocal scatterer is now decomposed as:

(21) $$S=S_{rec}={\alpha \hat{s}}_{a}+\beta {\hat{s}}_{b}+\gamma {\hat{s}}_{c}$$

where

(22) $$\alpha =\frac{{S_{HH}+S}_{VV}}{\sqrt{2}},\beta =\frac{{S_{HH}-S}_{VV}}{\sqrt{2}},\gamma =\sqrt{2}S_{HV}$$

Ultimately, the reciprocal scatterer is expressed as follows:

(23) $$s_{rec}=\alpha \frac{1}{\sqrt{2}}\left[\begin{array}{c} 1\\ 0\\ 0\\ 1 \end{array}\right]+\beta \frac{1}{\sqrt{2}}\left[\begin{array}{c} 1\\ 0\\ 0\\ -1 \end{array}\right]+\gamma \frac{1}{\sqrt{2}}\left[\begin{array}{c} 0\\ 1\\ 1\\ 0 \end{array}\right]$$

The reciprocal scatterer is considered symmetric if it possesses an axis of symmetry perpendicular to the radar Line of Sight (LOS), or if there’s a rotation angle $\psi_{c}$ that nullifies the projection of $S_{rec}$ on the antisymmetric component $S_{c}$. When such an angle is present, the symmetric aspect of the reciprocal scatterer achieves its utmost magnitude. The rotation angle $\psi_{c}$ represents the orientation angle of the scatterer. The maximum symmetric component of the reciprocal scatterer is described as:

(24) $$S_{sym}^{max}={\alpha S}_{a}+\varepsilon S_{b}$$

with

(25) $$\varepsilon =\beta cos\left(\chi \right)+\gamma sin\left(\chi \right)$$

and

(26) $$\tan\left(2\chi \right)=\frac{\beta \gamma^{*}+\beta^{*}\gamma }{\left({\left|\beta \right|}^{2}+{\left|\gamma \right|}^{2}\right)}$$

when *β* ≠γ. Alternatively, if *β* = γ then χ=0. The orientation angle of the scatterer can be found as follows:

(27) $$\psi =-\frac{1}{4}\chi ,\;\;\;\;\;-\pi \leq \chi \leq \pi$$

As for the degree of symmetry, it is expressed as the degree to which *S* deviates from $S_{sym}^{max}$, and it can be calculated as:

(28) $$\cos^{\;}\tau_{\mathrm{sym}}=\left|\frac{\left(S,\;S_{\mathrm{sym}}^{\max}\right)}{\left|\left|S\right|\right|\cdot \left|\left|S_{\mathrm{sym}}^{\max}\right|\right|}\right|,0\leq \tau_{sym}\leq \frac{\pi }{4}$$

The symbol ||…|| denotes the norm of a complex vector form, which aligns with the associated matrix.

In the case where $\tau_{sym}=0$, the scattering matrix represents a perfectly symmetric target. Conversely, if $\tau_{sym}=\frac{\pi }{4}$, the target that backscattered the radiation is regarded as asymmetric. Cameron delineates symmetry by categorizing any elementary scatterer with an angle $\tau_{sym}\leq \frac{\pi }{8}$ as symmetric, otherwise he considers it as asymmetric. Examples of asymmetric scattering matrices are the left and right helix, given by the equations:

(29) $$S_{lh}=\frac{1}{2}\left[\begin{array}{cc} 1 & i\\ i & -1 \end{array}\right]$$

(30) $$S_{rh}=\frac{1}{2}\left[\begin{array}{cc} 1 & -i\\ -i & -1 \end{array}\right]$$

The maximum symmetric component can be transformed into a normalized complex vector $\hat{\Lambda }\left(z\right)$ with z representing the complex parameter that ultimately dictates the scattering mechanism. The definition of the normalized complex vector $\;\hat{\Lambda }\left(z\right)$ is as follows:

(31) $$\hat{\Lambda }\left(z\right)=\frac{1}{\sqrt{1+{\left|z\right|}^{2}}}\left[\begin{array}{c} 1\\ 0\\ 0\\ z \end{array}\right],\;z\in ∁,\;\left|z\right|\leq 1$$

**Figure 5:**
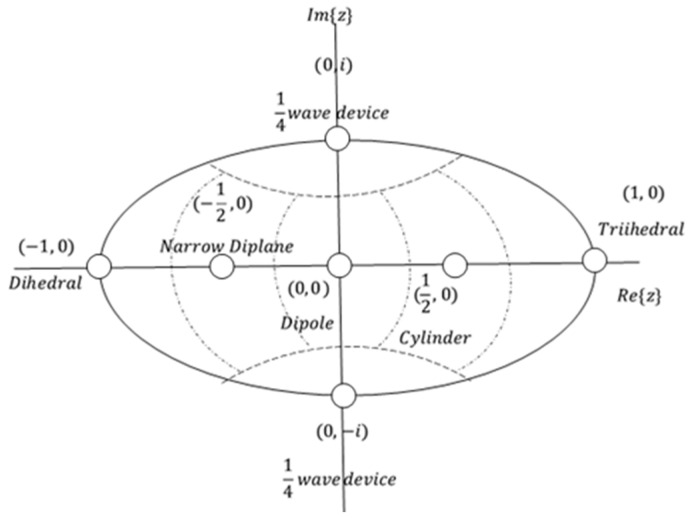
The arrangement of different elementary scattering mechanisms on Cameron’s Unit Disk visualization.

Table 1 provides detailed information on the complex vectors Λ(z) and their corresponding values of z for symmetric elementary scattering types. The parameter z’s range implies that the scattering matrix can be accurately depicted by a point situated on the unit disc within the complex plane. Figure 5 visually illustrates the positions of various elementary scattering mechanisms on the unit disk, accompanied by delineated regions representing their association with these scattering mechanisms. A crucial observation is that, in accordance with the values of *z* presented in Table 1, all elementary scatterers are situated along the diameter of the unit disk, with the exception of the ¼ wave devices, which specifically reside on the imaginary axis.

To assess the scattering characteristics of an unidentified scatterer *z*, Cameron devised a novel distance metric as follows:

(32) $$d\left(z,z_{ref}\right)=\cos^{-1}\left[\frac{\max\left(\left|1+zz_{ref}^{*}\right|,\left|z+z_{ref}^{*}\right|\right)}{\sqrt{\left(1+{\left|z\right|}^{2}\right)\left(1+{\left|z_{ref}\right|}^{2}\right)\;\;}}\right]$$

This expression provides a measure of similarity with each of the elementary scattering mechanisms referenced in Table 1.

In summary, Cameron’s CTD can be compactly formulated as:

(33) $$s=\alpha [cos\theta_{rec}+({cos\tau }_{sym}{\hat{s}}_{sym}^{max}+{sin\tau }_{sym}{\hat{s}}_{sym}^{min})+{sin\theta }_{rec}{\hat{s}}_{nonrec}]$$

where *α* is the total span of the backscattering matrix *S*, $\theta_{rec}$ determines the degree to which the scatterer deviates from the reciprocal space and $\tau_{sym}$ determines the symmetry degree of the scatterer. Ultimately, leveraging the maximum symmetric component allows for unambiguous extraction of information regarding the scatterer’s orientation angle, degree of symmetry, and dominant scattering mechanism.

**Figure 6:**
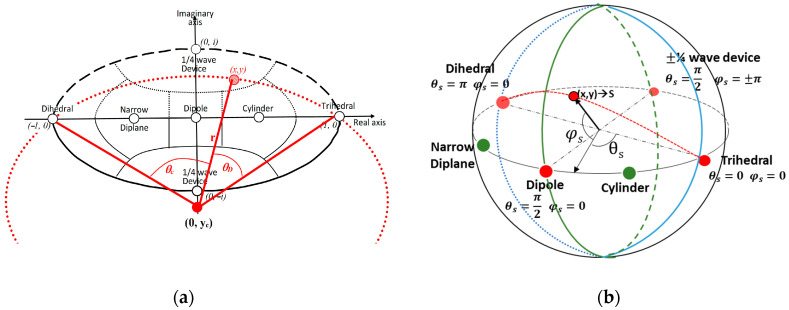
Depiction of the transformation process for a point (x,y) on the unit disk onto the unit sphere, along with the corresponding locations assumed by the symmetric scattering mechanisms. (**a**) A side view representation of the unit disk, where the diameter encompassing the elementary scatterers is projected onto the front half of the equator of the unit sphere. (**b**) The ¼ Wave Devices are specifically positioned on the rear side of the equator of the sphere during the mapping procedure.

Cameron et al. in [22] investigated the characteristics of conservative symmetric scattering mechanisms, emphasizing the preference for a closed surface over a complex disk. The proposed optimal configuration involves a symmetric space represented by the unit sphere, achieved by linking conjugate pairs along the rim of the unit disk. This conceptualization was thoroughly illustrated through a mapping procedure outlined in [22], with its visual depiction showcased in Figure 6a,b. To elaborate, in this novel topology, they established a direct association between each point (*x*,*y*) within the unit disk and a circular arc, denoted as a *a*(*x*,*y*) on the unit sphere that encompasses the points (−1,0), (*x*,*y*) and (1,0). Notably, for points (*x*,*y*) not situated on the rim of the disk, the arc length is less than *π*. In such instances, the arc is “stretched” to attain a length equal to π, becoming part of a great circle. The mapping is visually clarified by associating each point (*x*, *y*) with a semi-circle, strategically positioned tangent to the sphere’s surface. The initial position (*x*,*y*) on the unit disk determines the latitude $\varphi_{s}$ and longitude $\theta_{s}$ of the corresponding point on the unit sphere, with the spherical coordinates $\theta_{s}$ and $\varphi_{s}$ given by:

(34) $$\theta_{s}\left(x,y\right)=\left\{\begin{array}{c} \kappa \theta_{D}=\pi \frac{{sin}^{-1}\left(\frac{\sqrt{{\left(1-x\right)}^{2}+y^{2}}}{2r}\right)}{\sin^{-1}\left(\frac{1}{r}\right)\;\;\;\;}\;\;\;y\neq 0\;\\ \frac{\pi }{2}\left(1-x\right),\;\;\;\;\;\;\;\;\;\;y=0 \end{array}\right.$$

(35) φsx,y=4sin−1r−yc2,  y>0  0,       y=0 −4sin−1r−yc2,  y<0

where

(36) $$\kappa \left(x,y\right)=\frac{\pi }{\theta_{c}}=\frac{\pi }{2\sin^{-1}\left(\frac{1}{r}\right)\;\;\;},\;\;\;\;\;\;\;\;\;\;and\;\;\;\;y\neq 0$$

and

(37) $$\theta_{D}\left(x,y\right)=2\;{sin}^{-1}\left(\frac{\sqrt{{\left(1-x\right)}^{2}+y^{2}}}{2r}\right),\;\;\;\;and\;\;\;\;y\neq 0$$

Consequently, the updated configuration of the symmetrical scatterer unit as described in [18], is illustrated in Figure 6b. Additionally, the spatial distance measure, denoted as *d*, between a test elementary scattering mechanism *z* and each of the reference ones from Table 1, is now expressed in a more intuitive form akin to Equation (32):

(38) $$d\left(z,z_{ref}\right)=\sin^{-1}\left(\min\left[d_{-}\left(z{,z}_{ref}\right),\;d_{*}\left(z,z_{ref}\right)\right]\right)$$

with

(39) $$d_{-}\left(z{,z}_{ref}\right)=\sqrt{\frac{{\left|z{-z}_{ref}\right|}^{2}}{\left(1+{\left|z\right|}^{2}\right)\left(1+{\left|z_{ref}\right|}^{2}\right)}}$$

(40) $$d_{*}\left(z{,z}_{ref}\right)=\sqrt{\frac{{\left|z{-z}_{ref^{\;}}^{*}\right|}^{2}+(1-{\left|z\right|}^{2})(1-{\left|z_{ref}^{*}\right|}^{2})}{\left(1+{\left|z\right|}^{2}\right)\left(1+{\left|z_{ref}^{*}\right|}^{2}\right)}}$$

**Figure 7:**
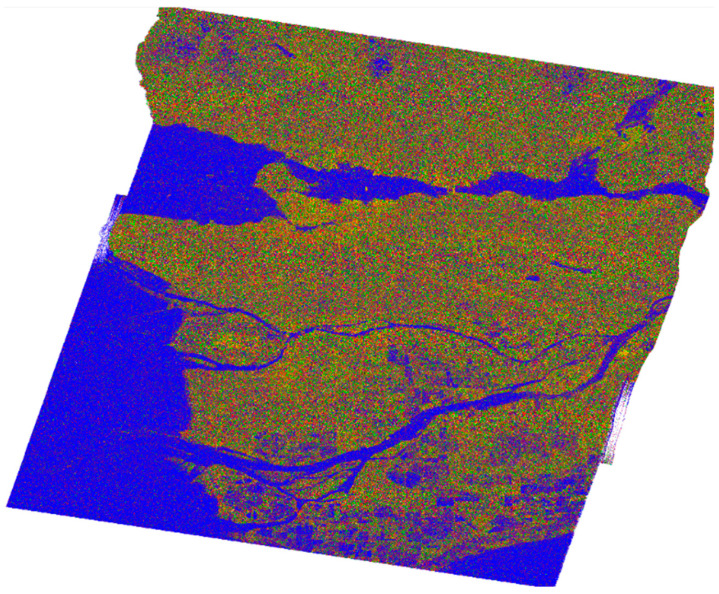
Color-coded PolSAR image of the broader area of Vancouver, based on the Cameron CTD.

The broader area of Vancouver, in color representation, based on Cameron’s decomposition is given in Figure 7, according to the color palette of Table 2.

As a coherent decomposition technique, Cameron’s approach has been exploited with remarkable results in target detection tasks. Specifically, Ringrose et al. [43] use Cameron’s method to discriminate ships from clutter based on the dominant scattering mechanism in each PolSAR cell. Moreover, it has been proved that the utilization of the features extracted based on Cameron’s approach by machine learning algorithms presents significant results in both detection and classification procedures. Namely, Kouroupis and Anastassopoulos [44] introduced a polarimetric CFAR ship detector in a Cameron-based Markov-ruled environment. G. Koukiou and V. Anastassopoulos utilized Markov chains to categorize different types of land cover. They employed Cameron’s decomposition to extract relevant features for this classification [45]. Furthermore, K. Karachristos et al. conducted a study involving the use of hidden Markov models in combination with Cameron’s scattering technique for supervised land cover classification [46].

In conclusion, coherent target decomposition approaches have exhibited considerable success in target detection procedures, particularly in homogeneous environments. These methods offer a wealth of information about the backscattering behavior of targets within polarimetric cells, presenting clear and physically interpretable insights.

However, the limitation in the number of elementary scattering mechanisms they provide warrants a more thorough investigation. Exploring a broader array of elementary scattering mechanisms would enhance the diversity of physical interpretations. Furthermore, a comprehensive study is needed to delve into the impact of varying data quality across different frequency bands and environmental conditions. This analysis would shed light on the specific circumstances under which each method proves to be more accurate, contributing to a nuanced understanding of their strengths and weaknesses. The key information is succinctly summarized in the following table (Table 3).

## 4. Model-Based Decomposition

One of the most commonly employed categories of polarimetric decomposition techniques for PolSAR data are the model-based methods. These methods are built upon the underlying physical scattering models of microwaves. In these algorithms, the covariance or coherency matrix is decomposed into multiple components that correspond to various physical scattering mechanisms. The model-based decomposition methods were initially introduced through the three-component scattering model for SAR data presented by A. Freeman and S. L. Durden [24].

### 4.1. Freeman–Durden Decomposition

The Freeman–Durden decomposition examines the polarization covariance matrix, requiring no ground truth measurements, and divides it into three distinct components:

(a) The canopy scatter from a cloud of randomly oriented dipoles or volume.
(b) The even or double bounce scatter from a pair of orthogonal surfaces with different dielectric constants and
(c) The Bragg scatter from a moderately rough surface.

This composite scattering model is used to describe the polarimetric backscatter from naturally occurring scatterers [24]. Concerning the first component of canopy scattering, it is assumed to be the radar echo from a cloud of randomly oriented, very thin, cylinder-like scatterers. According to the authors [24], the canopy scattering can be represented by the following scattering matrix, expressed in the orthogonal linear (*H*,*V*) basis, in standard orientation:

(41) $$S=\left[\begin{array}{cc} S_{\upsilon } & 0\\ 0 & S_{h} \end{array}\right]$$

It should be noted that the polarization scattering matrix (shown in Equation (41)) utilized by Freeman and Durden in their original paper [24] differs from the one defined in Equation (1) that is widely employed. The distinction lies in the rotated elements, but this variation does not yield different results in terms of implementation. Therefore, the covariance matrix that will be used later differs from the one defined in Equation (6).

It is possible to assume that the scatterers are positioned randomly with respect to the radar’s line of sight and at an angle of *φ* from the vertical polarization direction. To determine the scattering matrix of a specific scatterer under such circumstances, rotation operators can be applied to align the vertical direction with the scatterer’s standard orientation. Following this, the scattered field can be computed and then rotated back to the radar’s coordinate system. The equation below provides a means of expressing the scattering matrix in the radar’s coordinate system in terms of the scattering matrix in the scatterer’s coordinate system:

(42) $$\begin{array}{l} \left[\begin{array}{cc} S_{VV} & S_{VH}\\ S_{HV} & S_{HH} \end{array}\right]=R\left(\varphi \right)\left[\begin{array}{cc} S_{\upsilon } & 0\\ 0 & S_{h} \end{array}\right]R\left(-\varphi \right)\\ \;=\left(\begin{array}{cc} S_{h}\sin^{2}\varphi +S_{\upsilon }\cos^{2}\varphi & (S_{\upsilon }-S_{h})cos\varphi \;sin\varphi \\ (S_{\upsilon }-S_{h})cos\varphi \;sin\varphi & S_{h}\cos^{2}\varphi +S_{\upsilon }\sin^{2}\varphi \end{array}\right) \end{array}$$

where *R*(*φ*) is the rotation matrix:

(43) $$R\left(\varphi \right)=\left(\begin{array}{cc} cos\varphi & sin\varphi \\ -sin\varphi & cos\varphi \end{array}\right)$$

It is worth mentioning that the radars transmitting and receiving coordinate systems are identical, leading to a symmetric scattering matrix as the equation $S_{HV}=S_{VH}$ holds. Therefore, the covariance matrix as formulated by Freeman–Durden, considering the rotated elements with respect to the normal form of Equation (6), reduces to a 3 × 3 matrix without any loss of information:

(44) $${\langle \left[C_{3}\right]\rangle }_{v}=\left[\begin{array}{ccc} \langle {\left|S_{VV}\right|}^{2}\rangle & \sqrt{2\langle S_{VV}S_{VH}^{*}\rangle } & \langle S_{VV}S_{HH}^{*}\rangle \\ \sqrt{2\langle S_{VH}S_{VV}^{*}\rangle } & 2\langle {\left|S_{VH}\right|}^{2}\rangle & \sqrt{2\langle S_{VH}S_{HH}^{*}\rangle }\\ \langle S_{HH}S_{VV}^{*}\rangle & \sqrt{2\langle S_{HH}S_{VH}^{*}\rangle } & \langle {\left|S_{HH}\right|}^{2}\rangle \end{array}\right]$$

Taking into consideration that the probability density function for scatterers orientation is *p*(*φ*) and the expected value of any function *f*(*φ*) is:

(45) $$\langle f\rangle =\int_{0}^{2\pi }f\left(\varphi \right)p\left(\varphi \right)d\varphi$$

the elements of the covariance matrix according to the authors [24] are defined as follows:

(46) $$\langle {\left|S_{HH}\right|}^{2}\rangle =a_{1}{\left|S_{h}\right|}^{2}+2a_{2}Re\left(S_{h}S_{\upsilon }^{*}\right)+{a_{3}\left|S_{\upsilon }\right|}^{2}$$

(47) $$\langle {\left|S_{VV}\right|}^{2}\rangle =a_{1}{\left|S_{\upsilon }\right|}^{2}+2a_{2}Re\left(S_{h}S_{\upsilon }^{*}\right)+{a_{3}\left|S_{h}\right|}^{2}$$

(48) $$\langle {\left|S_{HV}\right|}^{2}\rangle =a_{2}{\left|S_{\upsilon }\right|}^{2}-2a_{2}Re\left(S_{h}S_{\upsilon }^{*}\right)+{a_{2}\left|S_{h}\right|}^{2}$$

(49) $$\langle S_{HH}S_{VV}^{*}\rangle =(a_{1}+a_{3})Re\left(S_{h}S_{\upsilon }^{*}\right)+a_{2}({\left|S_{h}\right|}^{2}+{\left|S_{\upsilon }\right|}^{2})+i(a_{1}-a_{3})Im(S_{h}S_{\upsilon }^{*})$$

(50) $$\langle S_{HH}S_{HV}^{*}\rangle =a_{4}(S_{h}S_{\upsilon }^{*}-{\left|S_{h}\right|}^{2})+a_{5}({\left|S_{\upsilon }\right|}^{2}-S_{\upsilon }S_{h}^{*})$$

(51) $$\langle S_{HV}S_{VV}^{*}\rangle =a_{4}({\left|S_{\upsilon }\right|}^{2}-S_{h}S_{\upsilon }^{*})+a_{5}(S_{\upsilon }S_{h}^{*}-{\left|S_{h}\right|}^{2})$$

where

(52) $$a_{1}\equiv \int_{0}^{2\pi }\cos^{4}\varphi \;p(\varphi )d\varphi$$

(53) $$a_{2}\equiv \int_{0}^{2\pi }\cos^{2}\varphi \;\sin^{2}\varphi \;p(\varphi )d\varphi$$

(54) $$a_{3}\equiv \int_{0}^{2\pi }\sin^{4}\varphi \;p(\varphi )d\varphi$$

(55) $$a_{4}\equiv \int_{0}^{2\pi }\cos^{3}\varphi \;sin\varphi \;p(\varphi )d\varphi$$

(56) $$a_{5}\equiv \int_{0}^{2\pi }cos\varphi \;\sin^{3}\varphi \;p(\varphi )d\varphi$$

For thin cylindrical scatterers, $S_{\upsilon }$ equals 1 and $S_{h}$ equals 0. Moreover, according to the assumption of a uniform orientation distribution *p*(*φ*), which implies that $a_{1}=a_{3}=\frac{3\pi }{4}$, $a_{2}=\frac{\pi }{4}$ and $a_{4}=a_{5}=0$, the covariance matrix of the ensemble of the very thin, cylinder-like scatterers can be modeled by:

(57) $$\;\;\;\;\;\;\;\;\;\;\;\;\;{\langle \left[C_{3}\right]\rangle }_{v}=\left[\begin{array}{ccc} \langle {\left|S_{VV}\right|}^{2}\rangle & \sqrt{2\langle S_{VV}S_{VH}^{*}\rangle } & \langle S_{VV}S_{HH}^{*}\rangle \\ \sqrt{2\langle S_{VH}S_{VV}^{*}\rangle } & 2\langle {\left|S_{VH}\right|}^{2}\rangle & \sqrt{2\langle S_{VH}S_{HH}^{*}\rangle }\\ \langle S_{HH}S_{VV}^{*}\rangle & \sqrt{2\langle S_{HH}S_{VH}^{*}\rangle } & \langle {\left|S_{HH}\right|}^{2}\rangle \end{array}\right]=f_{v}\left[\begin{array}{ccc} 1 & 0 & \frac{1}{3}\\ 0 & \frac{2}{3} & 0\\ \frac{1}{3} & 0 & 1 \end{array}\right]$$

where $f_{v}$ corresponds to the contribution of the volume (or canopy) scattering to the ${\left|S_{VV}\right|}^{2}$ component. The covariance matrix ${\langle \left[C_{3}\right]\rangle }_{v}$ is of rank 3, indicating that the scattering behavior cannot be characterized by a single scattering matrix of a pure target.

The second component of the Freeman–Durden decomposition approach, known as even or double bounce, is modeled by scattering from a dihedral corner reflector, where the reflector surfaces can be made of different dielectric materials. Therefore, the scattering matrix is modeled using reflection coefficients $R_{th}$ and $R_{tv}$ for horizontal and vertical polarizations on the vertical surface, and $R_{gh}$ and $R_{gv}$ for the same polarizations on the horizontal surface. In addition, the model is generalized by the two-phase components $e_{\;}^{j2\gamma_{\upsilon }}$ and $e_{\;}^{j2\gamma_{h}}$, where γ is a complex number that represents any attenuation and phase change of the vertically and horizontally polarized waves as they propagate from the radar to the ground and back again. As a result, the scattering matrix of the general even-bounce case can be determined as follows:

(58) $$S=\left[\begin{array}{cc} e_{\;}^{j2\gamma_{v}}R_{gv}R_{tv} & 0\\ 0 & e_{\;}^{j2\gamma_{h}}R_{gh}R_{th} \end{array}\right]$$

After normalization with respect to the $S_{VV}$ component, the corresponding covariance matrix can be written as:

(59) $${\langle \left[C_{3}\right]\rangle }_{d}=f_{d}\left[\begin{array}{ccc} 1 & 0 & \alpha^{*}\\ 0 & 0 & 0\\ \left|\alpha \right| & 0 & {\left|\alpha \right|}^{2} \end{array}\right]$$

where

(60) $$\alpha =e^{2j\left(\gamma_{h}{-\gamma }_{\upsilon }\right)}\frac{\left(R_{th}R_{gh}\right)}{R_{t\upsilon }R_{g\upsilon }}$$

(61) $$f_{d}={\left|R_{tv}R_{gv}\right|}^{2}$$

The covariance matrix ${\langle \left[C_{3}\right]\rangle }_{d}$ is of rank 1, indicating that the scattering behavior can be represented by a pure target.

A first-order Bragg model is employed for the third and last component, with the following scattering matrix:

(62) $$S=\left[\begin{array}{cc} R_{v} & 0\\ 0 & R_{h} \end{array}\right]$$

Hence, the covariance matrix is:

(63) $${\langle \left[C_{3}\right]\rangle }_{s}=f_{s}\left[\begin{array}{ccc} 1 & 0 & \beta^{*}\\ 0 & 0 & 0\\ \left|\beta \right| & 0 & {\left|\beta \right|}^{2} \end{array}\right]$$

where $f_{s}$ is defined as the contribution of the surface contribution to the ${\left|S_{VV}\right|}^{2}$ component, with

(64) $$f_{s}={\left|R_{v}\right|}^{2}$$

(65) $$\beta =\frac{R_{v}}{R_{h}}$$

In a similar manner to the previous case of even bounce scattering mechanism, the covariance matrix ${\langle \left[C_{3}\right]\rangle }_{s}$ of the first order Bragg surface exhibits a rank of 1. Consequently, it can be fully represented by the scattering mechanism presented at Equation (63).

By making the assumption that the volume, even bounce and surface scatterer components are uncorrelated, then the second-order statistics for the overall scattering behavior can be obtained by the sum of each individual scattering mechanism. Therefore, the model for the total backscatter is:

(66) $${\left[C\right]}_{3}={\langle \left[C_{3}\right]\rangle }_{v}+{\left[C_{3}\right]}_{d}+{\left[C_{3}\right]}_{s}$$

(67) $$\begin{array}{l} {\left[C\right]}_{3}=\left[\begin{array}{ccc} \langle {\left|S_{VV}\right|}^{2}\rangle & \sqrt{2\langle S_{VV}S_{VH}^{*}\rangle } & \langle S_{VV}S_{HH}^{*}\rangle \\ \sqrt{2\langle S_{VH}S_{VV}^{*}\rangle } & 2\langle {\left|S_{VH}\right|}^{2}\rangle & \sqrt{2\langle S_{VH}S_{HH}^{*}\rangle }\\ \langle S_{HH}S_{VV}^{*}\rangle & \sqrt{2\langle S_{HH}S_{VH}^{*}\rangle } & \langle {\left|S_{HH}\right|}^{2}\rangle \end{array}\right]\\ \;\;\;\;\;\;\;=\left[\begin{array}{ccc} \;f_{v}+f_{d}+f_{s} & 0 & \frac{f_{v}}{3}+f_{d}\alpha^{*}+f_{s}\beta^{*}\\ 0 & \frac{f_{v}}{3} & 0\\ \;\;\frac{f_{v}}{3}+f_{d}\alpha +f_{s}\beta & 0 & f_{v}+f_{d}{\left|\alpha \right|}^{2}+f_{s}\left|\beta \right| \end{array}\right] \end{array}$$

This model produces four equations in five unknowns. However, neither the surface nor the even bounce scattering mechanism contributes to the *HV* term, therefore, it is possible to calculate the volume contribution $f_{v}$ directly and then subtract the ${\left|S_{HH}\right|}^{2}$, ${\left|S_{VV}\right|}^{2}$, $S_{HH}S_{VV}^{*}$ terms, leaving the three equations:

(68) $$\langle {\left|S_{HH}\right|}^{2}\rangle ={f_{s}{\left|\beta \right|}^{2}+f_{d}{\left|\alpha \right|}^{2}}_{\;}$$

(69) $$\langle {\left|S_{VV}\right|}^{2}\rangle ={f_{s}+f_{d}}_{\;}$$

(70) $$\langle S_{HH}S_{VV}^{*}\rangle ={f_{s}\beta +f_{d}\alpha }_{\;}$$

If one of the unknowns is fixed, a solution can be determined. Van Zyl [26] proposes that the real part of $\langle S_{HH}S_{VV}^{*}\rangle$ can determine whether double bounce scattering, or surface scattering predominates in the residual. If $Re\left(\langle S_{hh}S_{vv}^{*}\rangle \right)\geq 0$, surface scattering is predominant, and the parameter α is set to α=−1. If $Re\left(\langle S_{hh}S_{vv}^{*}\rangle \right)$ is negative, double-bounce scattering is deemed dominant, and the parameter β is set to β=1. The contributions $f_{s}$ and $f_{d}$, as well as the parameters α, b, can be estimated from the residual radar measurements. Finally, the contribution of each scattering mechanism to the span can be estimated:

(71) $$Span={\left|S_{HH}\right|}^{2}+2{\left|S_{HV}\right|}^{2}+{\left|S_{VV}\right|}^{2}=P_{s}+P_{d}+P_{v}$$

where

(72) $$P_{s}=f_{s}\left(1+{\left|\beta \right|}^{2}\right)$$

(73) $$P_{d}=f_{d}\left(1+{\left|\alpha \right|}^{2}\right)$$

(74) $$P_{v}={\frac{8}{3}f}_{v}$$

**Figure 8:**
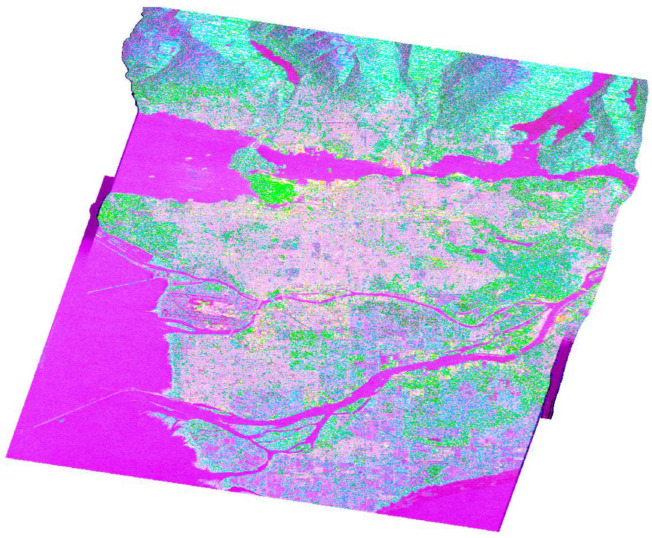
Color-coded PolSAR image of the broader area of San Francisco, based on the Freeman–Durden decomposition.

Therefore, the variables $P_{s}$, $P_{d}$ and $P_{v}$ can be utilized to produce an RGB image that encapsulates all the color-coded polarimetric information in a single image and is illustrated in Figure 8. In terms of color mapping, $P_{s}$ is represented by the blue channel, $P_{d}$ by the red channel and $P_{v}$ by the green channel. The color rendering was demonstrated using SNAP software 8.0.

The model-fitting approach of Freeman–Durden has a unique advantage, because it is established based on the fundamental principles of physics of radar scattering, rather than being a solely mathematical construction. This approach can be utilized to broadly identify the primary scattering mechanisms that produce the observed backscatter in polarimetric SAR data. The three-component scattering mechanism model has the potential to be valuable in creating features that distinguish various surface cover types and in determining the present state of those surfaces.

Although the three-model decomposition method can be utilized in most cases, it is subject to two significant constraints that limit its practicality. Firstly, the validity of the three components it relies upon may not always hold. Secondly, the accuracy of the results depends on the correlation coefficients $\langle S_{hh}S_{hv}^{*}\rangle =\langle S_{hv}S_{vv}^{*}\rangle =0$ used, which assume reflection symmetry. The first limitation restricts the use of the model to a specific group of scattering problems (initially intended by Freeman for terrain and forest backscattering applications) and may be inadequate when considering surface scattering with a non-zero entropy. However, the second assumption is more significant, as it applies to a broad range of scattering problems in scattering media exhibiting either reflection symmetry or rotation symmetry, even mixing both, referred to as azimuthal symmetry [47].

As already mentioned, Freeman and Durden’s decomposition is mainly efficient in terrain and forest backscattering applications. Thus, many studies use the three-component model to monitor and map rice crops [48,49], river ice cover [50], land use–land cover [51,52], and in studies on soil moisture [53,54].

### 4.2. Yamaguchi Decomposition

The three-component model introduced by Freeman and Durden [24] has been proven effective in the analysis of polarimetric data when the reflection symmetry condition is met. However, there are instances where certain areas in a SAR image may not conform to this condition. In such cases, Yamaguchi et al. [25] proposed a four-component scattering model in 2005, building upon the three-component model, by adding an extra term known as helix scattering power component, which corresponds to non-reflection symmetric cases where $\langle S_{HH}S_{HV}^{*}\rangle \neq 0$ and $\langle S_{HV}S_{VV}^{*}\rangle \neq 0$.

The helix component is more effective in complex urban areas and is useful for interpreting mostly man-made targets in heterogeneous regions like urban and suburban areas, while it is uncommon in natural distributed scattering. Cameron’s approach presented the polarimetric scattering matrices for the left and right helices in Equations (29) and (30). Therefore, the covariance matrices for the monostatic case where the reciprocity theorem holds $\left(S_{HV}=S_{VH}\right)$ and have the general form of:

(75) $${\langle \left[C_{3}\right]\rangle }_{v}=\left[\begin{array}{ccc} \langle {\left|S_{HH}\right|}^{2}\rangle & \sqrt{2\langle S_{HH}S_{HV}^{*}\rangle } & \langle S_{HH}S_{VV}^{*}\rangle \\ \sqrt{2\langle S_{HV}S_{HH}^{*}\rangle } & 2\langle {\left|S_{HV}\right|}^{2}\rangle & \sqrt{2\langle S_{HV}S_{VV}^{*}\rangle }\\ \langle S_{VV}S_{HH}^{*}\rangle & \sqrt{2\langle S_{VV}S_{HV}^{*}\rangle } & \langle {\left|S_{VV}\right|}^{2}\rangle \end{array}\right]$$

are given by:

(76) $${\left[C\right]}_{3l-helix}=\frac{f_{c}}{4}\left[\begin{array}{ccc} 1 & -j\sqrt{2} & -1\\ j\sqrt{2} & 2 & -j\sqrt{2}\\ -1 & j\sqrt{2} & 1 \end{array}\right]$$

(77) $${\left[C\right]}_{3r-helix}=\frac{f_{c}}{4}\left[\begin{array}{ccc} 1 & j\sqrt{2} & -1\\ -j\sqrt{2} & 2 & j\sqrt{2}\\ -1 & -j\sqrt{2} & 1 \end{array}\right]$$

where $f_{c}$ corresponds to the magnitude of the helix scattering component.

By considering the new term of the four-component scattering model, and for the sake of simplicity, the following definition of the scattering matrix adopted:

(78) $$S=\left[\begin{array}{cc} S_{HH} & S_{HV}\\ S_{VH} & S_{VV} \end{array}\right]=\left[\begin{array}{cc} a & c\\ c & b \end{array}\right]$$

Following the author’s methodology in [25], the polarimetric scattering matrix, rotated by an angle φ around the radar line of sight, is given by the following expression:

(79) $$\left[\begin{array}{cc} S_{hh} & S_{hv}\\ S_{vh} & S_{vv} \end{array}\right]=\left[\begin{array}{cc} cos\varphi & sin\varphi \\ -sin\varphi & cos\varphi \end{array}\right]\left[\begin{array}{cc} S_{HH} & S_{HV}\\ S_{VH} & S_{VV} \end{array}\right]\left[\begin{array}{cc} cos\varphi & -sin\varphi \\ sin\varphi & cos\varphi \end{array}\right]$$

The capital letters correspond to the original polarization measurements basis and to the measurable quantities while the small letters refer to the rotated coordinates. According to Yamaguchi’s original work [25] the covariance matrix elements are obtained via integration using a probability function $p\left(\varphi \right)$ according to:

(80) $$<S_{hh}^{*}S_{hh}>=\int_{0}^{2\pi }S_{hh}S_{hh}^{*}p\left(\varphi \right)d\varphi$$

The above also highlights the distinction between capital letters notation HV and the lowercase hv. HV denotes spatial ensemble averaging of spatial data, as previously mentioned, while hv corresponds to mathematical averaging defined by the integration expressions introduced in Equation (80).

The resultant terms are derived as follows:

(81) $$\langle {\left|S_{hh}\right|}^{2}\rangle =a_{1}{\left|a\right|}^{2}+a_{2}{\left|b\right|}^{2}+a_{3}{\left|c\right|}^{2}+2a_{4}Re\left(ab^{*}\right)+2a_{5}Re\left(ac^{*}\right)+2a_{6}Re(bc^{*})$$

(82) $$\langle {\left|S_{vv}\right|}^{2}\rangle =a_{2}{\left|a\right|}^{2}+{\left|b\right|}^{2}a_{1}+{\left|c\right|}^{2}a_{3}+{2a}_{4}Re\left(ab^{*}\right)-2a_{6}Re\left(ac^{*}\right)-2a_{5}Re(bc^{*})$$

(83) $$\langle {\left|S_{hv}\right|}^{2}\rangle =\frac{1}{4}{a_{3}\left|b-a\right|}^{2}+a_{7}{\left|c\right|}^{2}+a_{8}Re(c^{*}\left(b-a\right))$$

(84) $$\begin{array}{l} \langle S_{hh}S_{vv}^{*}\rangle =a_{4}({\left|a\right|}^{2}+{\left|b\right|}^{2})-{\alpha_{3}\left|c\right|}^{2}+a_{1}\left(ab^{*}\right)+a_{2}\left(a^{*}b\right)+a_{5}\left(b^{*}c-ac^{*}\right)\\ \;\;\;\;\;\;+a_{6}(a^{*}c-bc^{*}) \end{array}$$

(85) $$\begin{array}{l} \langle S_{hh}S_{hv}^{*}\rangle =a_{5}\frac{a\left(b^{*}-a^{*}\right)}{2}+a_{6}\frac{b\left(b^{*}-a^{*}\right)}{2}+a_{3}\frac{c\left(b^{*}-a^{*}\right)}{2}+a_{10}c^{*}a+a_{9}bc^{*}\\ \;\;\;\;\;\;+a_{8}{\left|c\right|}^{2} \end{array}$$

(86) $$\begin{array}{l} \langle S_{hv}S_{vv}^{*}\rangle =a_{6}\frac{a^{*}\left(b-a\right)}{2}+a_{5}\frac{b^{*}\left(b-a\right)}{2}-a_{3}\frac{c^{*}\left(b-a\right)}{2}+a_{9}ca^{*}+a_{10}b^{*}c\\ \;\;\;\;\;\;-a_{8}{\left|c\right|}^{2} \end{array}$$

where

(87) $$a_{1}\equiv \int_{0}^{2\pi }\cos^{4}\varphi \;p(\varphi )d\varphi$$

(88) $$a_{2}\equiv \int_{0}^{2\pi }\sin^{4}\varphi \;p(\varphi )d\varphi$$

(89) $$a_{3}\equiv \int_{0}^{2\pi }\sin^{2}2\varphi \;p(\varphi )d\varphi$$

(90) $$a_{4}\equiv \int_{0}^{2\pi }\sin^{2}\varphi \;\cos^{2}\varphi \;p(\varphi )d\varphi$$

(91) $$a_{5}\equiv \int_{0}^{2\pi }\cos^{2}\varphi \;sin2\varphi \;p(\varphi )d\varphi$$

(92) $$a_{6}\equiv \int_{0}^{2\pi }\sin^{2}\varphi \;sin2\varphi \;\;p(\varphi )d\varphi$$

(93) $$a_{7}\equiv \int_{0}^{2\pi }\cos^{2}2\varphi \;p(\varphi )d\varphi$$

(94) $$a_{8}\equiv \int_{0}^{2\pi }sin2\varphi \;cos2\varphi \;p(\varphi )d\varphi$$

(95) $$a_{9}\equiv \int_{0}^{2\pi }\sin^{2}\varphi \;cos2\varphi \;p(\varphi )d\varphi$$

(96) $$a_{10}\equiv \int_{0}^{2\pi }\cos^{2}\varphi \;cos2\varphi \;p(\varphi )d\varphi$$

In the case of uniform probability density function $p\left(\varphi \right)=\frac{1}{2\pi }$, the following simplified results are obtained:

(97) $$\langle {\left|S_{hh}\right|}^{2}\rangle =\langle {\left|S_{vv}\right|}^{2}\rangle =\frac{1}{8}{\left|a+b\right|}^{2}+\frac{1}{4}({\left|a\right|}^{2}+{\left|b\right|}^{2})+\frac{1}{2}{\left|c\right|}^{2}$$

(98) $$\langle S_{hh}^{\;}S_{vv}^{*}\rangle =\langle S_{hh}^{*}S_{vv}\rangle =\frac{1}{8}{\left|a+b\right|}^{2}+\frac{1}{2}Re\left(a^{*}b\right)-\frac{1}{2}{\left|c\right|}^{2}$$

(99) $$\langle {\left|S_{hv}\right|}^{2}\rangle =\frac{1}{8}{\left|a-b\right|}^{2}+\frac{1}{2}{\left|c\right|}^{2}$$

(100) $$\langle S_{hh}S_{hv}^{*}\rangle =\langle S_{hv}^{\;}S_{vv}^{*}\rangle =\frac{j}{2}Im(c^{*}(a-b))$$

The uniform distribution yields the volume scattering covariance matrix corresponding to randomly oriented dipole as it is defined by the Freeman–Durden Decomposition Equation (57):

(101) $${\left[C\right]}_{vol}^{\;}=\frac{1}{8}\left[\begin{array}{ccc} 3 & 0 & 1\\ 0 & 2 & 0\\ 1 & 0 & 3 \end{array}\right]$$

Regardless of the format used for the polarimetric scattering matrix, whether for a vertical or horizontal dipole, the result remains independent. In the same sense for the double-bounce scatterer the polarimetric scattering matrix is defined as:

(102) $${\left[C\right]}_{double}^{\;}=\frac{1}{4}\left[\begin{array}{ccc} 1 & 0 & -1\\ 0 & 2 & 0\\ -1 & 0 & 1 \end{array}\right]$$

while for the single-bounce (plate or sphere) the form is:

(103) $${\left[C\right]}_{surface}^{\;}=\frac{1}{2}\left[\begin{array}{ccc} 1 & 0 & 1\\ 0 & 0 & 0\\ 1 & 0 & 1 \end{array}\right]$$

Despite the scattering matrix being different for horizontal and vertical orientations, the covariance matrix remains the same. This advantage underscores the usefulness of the covariance matrix in target decomposition. Moreover, the forms above satisfy the condition $Trace\left[C\right]=1$, which implies that the total power for each scattering mechanisms is unity. The above covariance matrices are in line with the three-component model as was introduced by Freeman and Durden under the reflection symmetry condition $\langle S_{hh}S_{hv}^{*}\rangle \approx \langle S_{vv}^{\;}S_{hv}^{*}\rangle \approx 0$.

In addition, Yamaguchi et al. [25] proposed a modification for the probability density function for the volume scattering matrix according to the relative backscattering magnitudes between $\langle {\left|S_{HH}\right|}^{2}\rangle$ and $\langle {\left|S_{VV}\right|}^{2}\rangle$. In particular, a model employing a cloud of randomly oriented dipoles with a uniform probability function for the orientation angles is used to represent volume scattering by vegetated areas. However, due to the dominant presence of vertical structure for forests, tree trunks and branches, a new probability distribution function for vegetations was proposed:

(104) $$p\left(\varphi \right)=\left\{\begin{array}{c} \frac{1}{2}sin\varphi ,\;for\;0<\varphi <\pi \\ 0,\;for\;\pi <\varphi <2\pi \end{array}\right.\;with\;\int_{0}^{2\pi }p(\varphi )d\varphi$$

where φ is taken from the horizontal axis seen from the radar. In this sense, the integrals defined Equations (87)–(96) are formed as follows:

(105) $$a_{1}=\frac{3}{15},\;a_{2}=a_{3}=\frac{8}{15},\;a_{4}=\frac{2}{15},\;a_{5}=a_{6}=a_{8}=0,\;a_{9}=-\frac{6}{15},\;a_{10}=\frac{1}{15}$$

These results indicate that the volume scattering covariance matrix for the vertical-oriented dipole is formed as:

(106) $$S_{vol}^{vertical}=\left[\begin{array}{cc} 0 & 0\\ 0 & 1 \end{array}\right]\to {\left[C\right]}_{vol}^{vertical}=\frac{1}{15}\left[\begin{array}{ccc} 8 & 0 & 2\\ 0 & 4 & 0\\ 2 & 0 & 3 \end{array}\right]$$

While for the horizontal oriented dipole:

(107) $$S_{vol}^{horizontal}=\left[\begin{array}{cc} 1 & 0\\ 0 & 0 \end{array}\right]\to \;{\left[C\right]}_{vol}^{horizontal}=\frac{1}{15}\left[\begin{array}{ccc} 3 & 0 & 2\\ 0 & 4 & 0\\ 2 & 0 & 8 \end{array}\right]$$

The value of the above asymmetric forms lies on the fact that it can be adjusted to the measured data with $\langle {\left|S_{HH}\right|}^{2}\rangle \neq \langle {\left|S_{VV}\right|}^{2}\rangle$ according to the ratio $10log(\frac{\langle {\left|S_{VV}\right|}^{2}\rangle }{\langle {\left|S_{HH}\right|}^{2}\rangle })$. Specifically, in the PolSAR scene, the four-component model chooses one of the asymmetric forms (Equation (106) or Equation (107)) if the relative magnitude difference is greater than 2 dB. Conversely, for a difference within the range of ±2 dB (Table 4), the symmetric form, as defined in Equation (101).

By making the assumption that the volume, even bounce, surface, and helix scatterer components are uncorrelated, the second-order statistics of the overall scattering pattern can be derived by summing each individual scatterer. Therefore, the model representing the entirety of the backscatter is as follows:

(108) $${\left[C\right]}_{3}={\langle \left[C_{3}\right]\rangle }_{v}+{\left[C_{3}\right]}_{d}+{\left[C_{3}\right]}_{s}+{\left[C_{3}\right]}_{h}$$

(109) $$\begin{array}{c} {\left[C\right]}_{3}=\left[\begin{array}{ccc} \langle {\left|S_{HH}\right|}^{2}\rangle & \sqrt{2\langle S_{HH}S_{HV}^{*}\rangle } & \langle S_{HH}S_{VV}^{*}\rangle \\ \sqrt{2\langle S_{HV}S_{HH}^{*}\rangle } & 2\langle {\left|S_{HV}\right|}^{2}\rangle & \sqrt{2\langle S_{HV}S_{VV}^{*}\rangle }\\ \langle S_{VV}S_{HH}^{*}\rangle & \sqrt{2\langle S_{VV}S_{HV}^{*}\rangle } & \langle {\left|S_{VV}\right|}^{2}\rangle \end{array}\right]=\frac{f_{v}}{15}\left[\begin{array}{ccc} 8 & 0 & 2\\ 0 & 4 & 0\\ 2 & 0 & 3 \end{array}\right]+f_{d}\left[\begin{array}{ccc} {\left|\alpha \right|}^{2} & 0 & \alpha \\ 0 & 0 & 0\\ \alpha^{*} & 0 & 1 \end{array}\right]+\\ f_{s}\left[\begin{array}{ccc} {\left|\beta \right|}^{2} & 0 & \beta \\ 0 & 0 & 0\\ \beta^{*} & 0 & 1 \end{array}\right]+\frac{f_{c}}{4}\left[\begin{array}{ccc} 1 & \pm j\sqrt{2} & -1\\ \mp j\sqrt{2} & 2 & \pm j\sqrt{2}\\ -1 & 0 & 1 \end{array}\right] \end{array}$$

This model produces five equations in six unknowns α, β, $f_{v}$, $f_{d}$, $f_{s}$ and $f_{c}$. The parameters $f_{s}$ and $f_{d}$ are identical with those defined in the Freeman–Durden three-component decomposition, $f_{v}$ is modified for data in line with the ratio $10log(\frac{\langle {\left|S_{VV}\right|}^{2}\rangle }{\langle {\left|S_{HH}\right|}^{2}\rangle })$*,* and the $f_{c}$ term corresponds to the helix scattering power contribution.

The five equations with the six unknowns are the following:

(110) $$\langle {\left|S_{HH}\right|}^{2}\rangle ={f_{s}{\left|\beta \right|}^{2}+f_{d}{\left|\alpha \right|}^{2}}_{\;}+\frac{8}{15}f_{v}+\frac{f_{c}}{4}$$

(111) $$\langle {\left|S_{HV}\right|}^{2}\rangle =\frac{2}{15}f_{v}+\frac{f_{c}}{4}$$

(112) $$\langle {\left|S_{VV}\right|}^{2}\rangle ={f_{s}+f_{d}+\frac{3}{15}f_{v}+\frac{f_{c}}{4}}_{\;}$$

(113) $$\langle S_{HH}S_{VV}^{*}\rangle ={f_{s}\beta +f_{d}\alpha }_{\;}+\frac{2}{15}f_{v}-\frac{f_{c}}{4}$$

(114) $$\;\;\;\;\frac{1}{2}Im\left\{<S_{HH}S_{HV}^{*}>+<S_{HV}S_{VV}^{*}>\right\}=\frac{f_{c}}{4}$$

Since, the left-hand side of Equation (114) is a measurable quantity, it directly results in:

(115) $$P_{c}=f_{c}=2|Im\left\{\langle S_{HH}S_{HV}^{*}\rangle +\langle S_{HV}S_{VV}^{*}\rangle \right\}|$$

Consequently, the volume scattering coefficient $f_{v}$ can be calculated from Equation (111):

(116) $$f_{v}=\frac{15}{2}(\langle {\left|S_{hv}\right|}^{2}\rangle -\frac{f_{c}}{4})$$

The other four unknown variables in the remaining three equations can be obtained in the same way as presented by Freeman and Durden, according to which scattering is dominant in order to fix the values for α and β [24]. In conclusion, the scattering powers corresponding to surface, double bounce, volume, and helix are as follows:

(117) $$P_{s}=f_{s}\left(1+{\left|\beta \right|}^{2}\right),\;\;\;P_{d}=f_{d}\left(1+{\left|\alpha \right|}^{2}\right),\;\;\;\;P_{v}f_{v},\;\;\;\;\;P_{c}=f_{c}$$

(118) $$SPAN=P_{s}+P_{d}+P_{v}+P_{c}=\langle {\left|S_{HH}\right|}^{2}+2{\left|S_{HV}\right|}^{2}+{\left|S_{VV}\right|}^{2}\rangle$$

This model has been shown to be more accurate in decomposing PolSAR images in areas with non-symmetric scattering and has become widely used in applications. Therefore, it is important to recognize the presence of non-symmetric scattering areas and apply appropriate models, such as the four-component scattering model proposed by Yamaguchi et al. to achieve precise results. Many studies have utilized the four-component decomposition in disaster monitoring [55] and terrain classification [56,57] with emphasis in wetlands and glaciated terrains [58,59,60].

In considering potential future advancements in model-based decomposition techniques, an evident avenue involves their integration with stochastic machine learning algorithms to enable a more intrinsic exploration of the data. Furthermore, exploring data-level fusion approaches that leverage diverse datasets acquired from various satellite sources holds significant promise. Specifically, for Freeman–Durden decomposition, there is potential in addressing complex terrain and heterogeneous environments to enhance its applicability. In the case of Yamaguchi Decomposition, forthcoming research may focus on refining the methodology to better characterize specific target scenarios. Researchers might delve into methods to augment the decomposition’s sensitivity to nuanced variations in target structures, especially in the presence of interfering elements. Additionally, investigating its application across different frequency bands or on various satellite platforms could broaden its effectiveness within diverse remote sensing contexts. A summarization of the key points regarding Model-Based Decomposition Techniques is depicted in Table 5.

## 5. Eigenvector–Eigenvalue Decomposition

Eigenvector-based techniques are considered an important class of target decomposition algorithms and first introduced by Cloude’s pioneering work [30]. These techniques exploit the basis invariance of the eigenvalue problem by utilizing the eigenvalues of the 3 × 3 Hermitian averaged coherency $T_{3}$ matrix. These techniques assume the validity of reciprocity theorem $\left(S_{HV}=S_{VH}\right)$, which allows for reducing the dimensions of the coherency matrix from 4 × 4, to 3 × 3 as demonstrated in the analysis of Freeman–Durden three components model Equation (44) and Yamaguchi four components model Equation (57).

By computing the eigenvectors and eigenvalues of the coherency matrix, a diagonal form of the coherency matrix can be produced and be physically interpreted by statistically independent target vectors. According to this method, the coherency matrix can be decomposed as:

(119) $${\langle \left[T_{3}\right]\rangle }_{\;}=\left[U_{3}\right]\Sigma {\left[U_{3}\right]}^{-1}$$

where Σ is a 3 × 3 diagonal matrix with nonnegative real elements and $U_{3}=[u_{1},u_{2},u_{3}]$ denotes a 3 × 3 unitary matrix of the SU(3) group, where $u_{1},u_{2}$ and $u_{3}$ are three-unit orthogonal vectors.

Hence, a statistical model can be constructed that analyzes the $\langle \left[T_{3}\right]\rangle$ matrix into the sum of three independent scattering mechanisms:

(120) $${\langle \left[T_{3}\right]\rangle }_{\;}=\sum_{i=1}^{3}\lambda_{i}u_{i}u_{i}^{T*}$$

where $\lambda_{i}$ are real numbers corresponding to the eigenvalues of the coherency matrix and representing the statistical weights for the type of scattering that have been occurred and related with the unitary vector $u_{i}$. In case of only one non-zero eigenvalue, the coherency matrix $T_{3}$ represents a single scattering mechanism corresponding to a pure target. In contrast, when all eigenvalues are equal, the coherency matrix comprises of three orthogonal scattering mechanisms with equal amplitudes, indicating a random target. Otherwise, for non-zero and unequal eigenvalues the scattering behavior corresponds to partial targets. To analyze the polarimetric properties of such targets, it is necessary to study the distribution of eigenvalues and characterize each scattering mechanism.

### 5.1. H/A/a. Decomposition

Among the target decomposition techniques, Cloude and Pottier’s $H/A/\bar{\alpha }$ approach [30], first introduced in 1997, has attracted considerable attention. Based on eigenvector decomposition of the coherency matrix, Cloude and Pottier established the utilization of a new set of parameters consisting of entropy, anisotropy, and angle α. Thus, each of the three scattering mechanisms that the eigenvalue decomposition produces can be described with these parameters. The method implies that scattering within one resolution cell is caused by a limited number of repeatedly occurring events with a specific frequency. Such an assumption led to a Bernoulli process, where several statistical events from a set of random variables $X_{n}$ occur in a sequential order. After the eigenvalue decomposition, such an approach is exploited. Specifically, the analysis focuses on the 3 × 3 Hermitian matrix $\langle \left[T_{3}\right]\rangle$ utilizing the eigen decomposition theorem as mentioned previously in Equations (119) and (120) is:

(121) $${\langle \left[T_{3}\right]\rangle }_{\;}=\left[U_{3}\right]\left[\Sigma_{3}\right]{\left[U_{3}\right]}^{-1}$$

The 3 × 3 real, diagonal matrix $\left[\Sigma_{3}\right]$ contains the eigenvalues of ${\langle \left[C_{3}\right]\rangle }_{\;}$

(122) $$\left[\Sigma_{3}\right]=\left[\begin{array}{ccc} \lambda_{1} & 0 & 0\\ 0 & \lambda_{2} & 0\\ 0 & 0 & \lambda_{3} \end{array}\right]$$

where $\lambda_{1}>\lambda_{2}>\lambda_{3}>0$.

As already mentioned, the 3 × 3 unitary matrix $\left[U_{3}\right]$ contains the eigenvectors $u_{i}$ which are defined as:

(123) $$u_{i}={\left[cos\alpha_{i}e^{j\varphi_{i}}\;sin\alpha_{i}cos\beta_{i}e^{j{(\delta }_{i}+\varphi_{i})}\;sin\alpha_{i}cos\beta_{i}e^{j{(\gamma }_{i}+\varphi_{i})}\right]}^{T}$$

Based on these three orthogonal eigenvectors the following parameterization of the unitary matrix is obtained:

(124) $$\left[U_{3}\right]=\left[\begin{array}{ccc} cos\alpha_{1}e^{j\varphi_{1}} & cos\alpha_{2}e^{j\varphi_{2}} & cos\alpha_{3}e^{j\varphi_{3}}\\ sin\alpha_{1}{cos\beta_{1}e}^{j(\delta_{1}+\varphi_{1})} & sin\alpha_{2}{cos\beta_{2}e}^{j(\delta_{2}+\varphi_{2})} & sin\alpha_{3}{cos\beta_{3}e}^{j(\delta_{3}+\varphi_{3})}\\ sin\alpha_{1}{sin\beta_{1}e}^{j(\gamma_{1}+\varphi_{1})} & sin\alpha_{2}{sin\beta_{2}e}^{j(\gamma_{2}+\varphi_{2})} & sin\alpha_{3}{sin\beta_{3}e}^{j(\gamma_{3}+\varphi_{3})} \end{array}\right]$$

This parameterization in terms of column vectors with different parameters $\alpha_{i}$, $\beta_{i}$, $\gamma_{i}$, $\delta_{i}$ leads to a probabilistic interpretation of the scattering process. Due to the assumption that the scattering within each resolution cell generates a Bernoulli process, the eigenvalues $\lambda_{i}$ corresponds to the absolute frequency of the occurrence of the three different scattering mechanisms. Therefore, the probability of occurrence can be defined as:

(125) $$P_{i}=\frac{\lambda_{i}}{\sum_{i=1}^{3}\lambda_{i}}\;i=[1,2,3]$$

In this way, any target parameter *x* follows a random sequence, and the best estimation is given by the mean of this sequence:

(126) $$\tilde{x}=\sum_{i=1}^{3}P_{i}x_{i}$$

Accordingly, the parameters of the scattering process, excepting *φ* which is physically equivalent to an absolute target phase, can be defined as:

(127) $$\tilde{\alpha }=\sum_{i=1}^{3}P_{i}\alpha_{i}\;\;\tilde{\beta }=\sum_{i=1}^{3}P_{i}\beta_{i}\;\;\tilde{\gamma }=\sum_{i=1}^{3}P_{i}\gamma_{i}\;\;\tilde{\delta }=\sum_{i=1}^{3}P_{i}\delta_{i}$$

Replacing the parameters by the expected values, the mean unit vector can now be constructed:

(128) $$u_{i}=e^{j\varphi_{i}}{\left[cos\tilde{\alpha }\;sin\tilde{\alpha }cos\tilde{\beta }e^{j\tilde{\delta }}\;sin\tilde{\alpha }cos\tilde{\beta }e^{j\tilde{\gamma })}\right]}^{T}$$

The alpha $\tilde{\alpha }$ angle is the main parameter or identifying the dominant scattering mechanisms, as being roll invariant, while the three others β,  γ, and δ can be used to define the target polarization orientation angle.

Angle $\tilde{\alpha }\in [0,\frac{\pi }{2}]$ corresponds to the dominant scattering mechanisms:

- for $\tilde{\alpha }=0$ the target is a plate;
- for $\tilde{\alpha }=\frac{\pi }{4}$ the target is a dipole;
- for $\tilde{\alpha }=\frac{\pi }{2}$ the target is a dihedral.

Angles $\tilde{\beta }$, $\tilde{\gamma }$, and $\tilde{\delta }$ describes further properties of the target and their values change with the orientation of the target and that complicates the interpretation. These parameters will not be explained here.

The entropy parameter H, as defined by Von Neumann, indicates the level of statistical disorder or randomness. In particular, in the case of a pure target with only one non-zero eigenvalue ($\lambda_{1}\neq 0,\;\lambda_{2}=\lambda_{3}=0$) resulting from eigenvalue decomposition, the entropy is 0. However, for a distributed target where all eigenvalues are equal ${(\lambda }_{1}=\lambda_{2}=\lambda_{3})$, the entropy parameter reaches its maximum value of 1.

The majority of targets in polarimetric data lie between the two extreme cases, referred to previously. To quantify the level of statistical disorder, the polarimetric entropy H is calculated as follows:

(129) $$H=-\sum_{k=1}^{N}P_{k}\log_{N}\left(P_{k}\right)$$

where $P_{i}$ corresponds to the pseudo-probabilities obtained from the eigenvalues $\lambda_{i}$, N is the logarithm basis that must be equal to the polarimetric dimension (N=3 for the monostatic case and N=4 for the bistatic one). It is worth noting that the H parameter is both basis-invariant and roll invariant since the eigenvalues are rotational invariant.

Although polarimetric entropy H is a valuable measure for characterizing the randomness of scattering problems, the polarimetric Anisotropy parameter is introduced as a complement to H based on the two smallest eigenvalues:

(130) $$A=\frac{\lambda_{2}-\lambda_{3}}{\lambda_{2}+\lambda_{3}}$$

As entropy is invariant to rotation due to the rotational invariance of eigenvalues, the anisotropy is also invariant to rotation. Anisotropy is a measurement of the importance of the second and the third eigenvalues. This parameter is essential when the entropy H takes high values (greater than 0.7) in order to determine the different types of scattering process.

### 5.2. H/alpha. Feature Space

**Figure 9:**
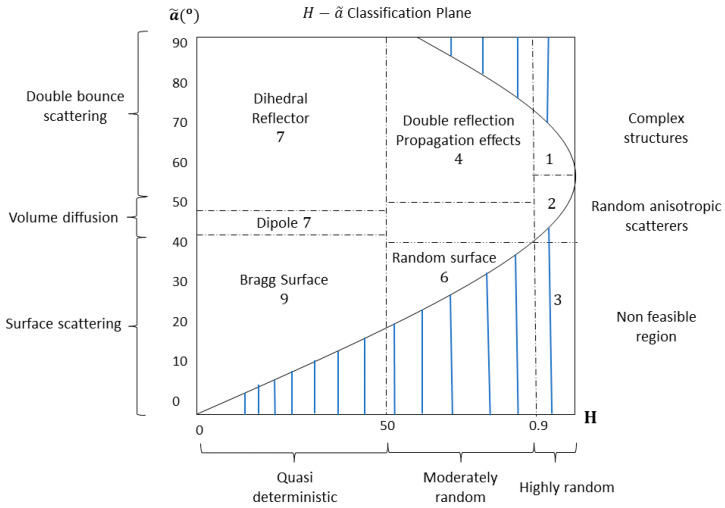
The two-dimensional H/alpha feature space.

By utilizing the parameters of entropy H and alpha angle $\tilde{\alpha }$, a 2D space is demonstrated, where each pixel is identified by these two parameters, providing information about the type of scattering. This space is divided into nine categories, as shown in Figure 9, with zone 3 being deemed non feasible.

**Figure 10:**
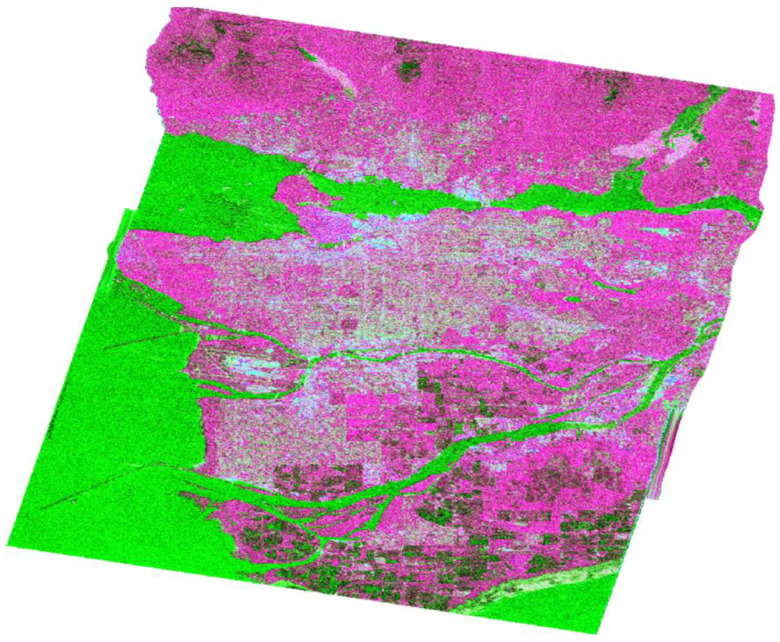
Color-coded PolSAR image of the broader area of Vancouver, based on the *H/A/a*-decomposition.

The three fundamental parameters of Cloude–Pottier decomposition can be utilized in classification tasks and color-coding, with Entropy represented by the color red, Anisotropy by green, and the alpha angle by blue (Figure 10).

The Cloude–Pottier Entropy-based decomposition is widely employed in various applications. The *H/A/a* approach has found utility in land cover classification [60,61] across diverse terrain types, including forested areas [62], snow-covered terrains [63,64], wetlands [65,66,67], and agricultural regions [68,69]. Although Cloude–Pottier is a non-coherent method, it has also been evaluated for target detection procedures despite not being endorsed for such applications [70,71].

In the realm of Cloude–Pottier Decomposition, potential future research avenues could delve into optimizing its performance in dynamic scenarios. Enhancing the decomposition’s robustness in the presence of temporal variations and evolving environmental conditions may be explored. Researchers could investigate strategies to improve the method’s adaptability to different imaging geometries and topographies. The key points of Cloude–Pottier Decomposition techniques are succinctly summarized in Table 6 for a quick reference.

## 6. The Double Scatterer Model

**Figure 11:**
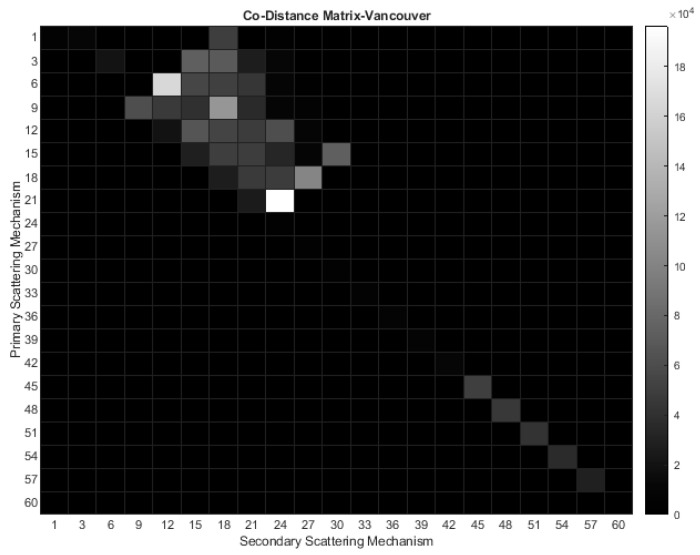
The Co-Distance Matrix, based on the Cameron CTD, illustrates the distances of the studied scatterer from its nearest and second-nearest counterparts. Measured in degrees according to Equation (32), the x and y axes depict these distances. The pixel intensity signifies the percentage of pixels associated with distance coordinates.

In Section 3.2. Cameron’s coherent Target Decomposition was presented. This decomposition encompasses a comprehensive toolchain for evaluating scattering mechanisms within a PolSAR cell. The toolchain incorporates a classification scheme that relies on the deterministic assumption of a single dominating scattering mechanism occurring in each PolSAR cell, thereby producing reliable results. However, the representation based on only one dominating elementary scattering center raises questions. Firstly, in real-world scenarios, many objects do not exclusively correspond to the elementary scattering mechanism set presented by Cameron. Secondly, the distance measure used in Equation (32) to identify the closest scatterer also assigns significant closeness to other mechanisms. This contrasts with the deterministic nature of the method, which implies that each PolSAR cell includes a dominant scattering. Consequently, the contribution of more than one elementary scattering to the scattering behavior of PolSAR cells should be considered. To establish the aforementioned inadequacy regarding the metric distance that determines the dominant scattering mechanism and to assess the newly proposed information extraction procedure, a series of experiments were conducted utilizing Fully Polarimetric datasets. By computing the distance from both the first and the second nearest scattering mechanism according to Cameron’s approach, the evaluation of Cameron’s classification scheme is shown. Thus, the results were demonstrated by means of co-occurrence matrices, as depicted in Figure 11. In this depiction, the measurements along the two axes are presented in degrees, as outlined in Equation (32). The X-values represent the distance from the second-nearest scattering mechanism, while the Y-values signify the distance from the closest scatterer.

The choice of using intervals/bins of 3 degrees was made based on the assumption that it provides a reliable range for drawing conclusions. Additionally, the maximum value for both the x and y axes, which correspond to the distance of the PolSAR cell under examination from the secondary and primary fundamental scattering mechanisms, respectively, is set to 60 degrees. This value was selected as none of the calculations yielded higher numbers during the analysis. The plots illustrate that all data points fall within the upper triangle of the co-distance matrices, indicating that the distance from the first elementary scatterer is typically shorter than from the second, as anticipated. However, the key observation is that a majority of points cluster around specific regions near the diagonal. This suggests that when the algorithm assigns the primary elementary scattering mechanism for each PolSAR cell, the disparity in distance from the second option is typically insignificant, resulting in ambiguous classification. Calculations reveal that slightly over 50% of PolSAR cells in both datasets are categorized into one of the eight elementary scattering mechanisms proposed by Cameron, with the difference in angles between the first and second nearest scattering mechanisms remaining under 9 degrees. Furthermore, around 30% of cells align with Cameron’s classification approach, displaying only a 3-degree distinction between the two predominant scatterers. These percentages would be even higher if the examined areas did not include regions that are inherently homogeneous, such as sea and water areas. As observed, even a slight discrepancy in the metric distance between the closest scattering mechanism and the adjacent secondary scatterer is adequate to categorize the former as predominant.

Moreover, in real-world scenarios, numerous objects do not align exclusively with the elementary scatterers outlined in Cameron’s decomposition (Table 2), leading to an uncertain evaluation of the SAR pixel’s scattering properties [72]. Consequently, relying solely on a single dominant scatterer can result in diminished discrimination.

To tackle these issues, the methodology presented by the Double Scatterer Model [33] adopts an information extraction approach that aims to explore the information content from both the dominant scattering mechanism and the second most influential scattering mechanism. Specifically, we employ a modification of the geometric topology proposed by Cameron and Rais [22] and introduce a method to represent each PolSAR cell with the two most dominant fundamental scattering mechanisms, along with their respective percentages of contribution or weights.

The Double Scatterer Model can be considered as an extension of Cameron’s coherent decomposition, which has been extensively analyzed. In this model, each PolSAR cell is interpreted using a pair of fundamental scattering mechanisms. The objective of the proposed method is to extract a maximum amount of polarimetric information from each PolSAR cell. The methodology comprises the following sequential steps:

1. For each PolSAR cell, the corresponding polarimetric scattering matrix is utilized following Cameron’s stepwise algorithm to calculate the complex parameter z. If the criteria of reciprocity and symmetry are satisfied, the maximum symmetric component of the scattering matrix can be defined as follows:

(131) $$S_{sym}^{max}=Ae^{j\varphi }R(\psi )\hat{\Lambda }\left(z\right)$$

where A denotes the amplitude of the scattering matrix, φ the absolute phase, ψ corresponds to the scatterer orientation angle and normalized complex vector $\hat{\Lambda }(z)$ is given by:

(132) $$\hat{\Lambda }\left(z\right)=\frac{1}{\sqrt{1+{\left|z\right|}^{2}}}\left[\begin{array}{c} 1\\ 0\\ 0\\ z \end{array}\right],z\in C,\left|z\right|\leq 1$$

As previously mentioned, the real and imaginary components of z are used to determine a corresponding point on the complex unit disk, following Cameron’s algorithm.

**Figure 12:**
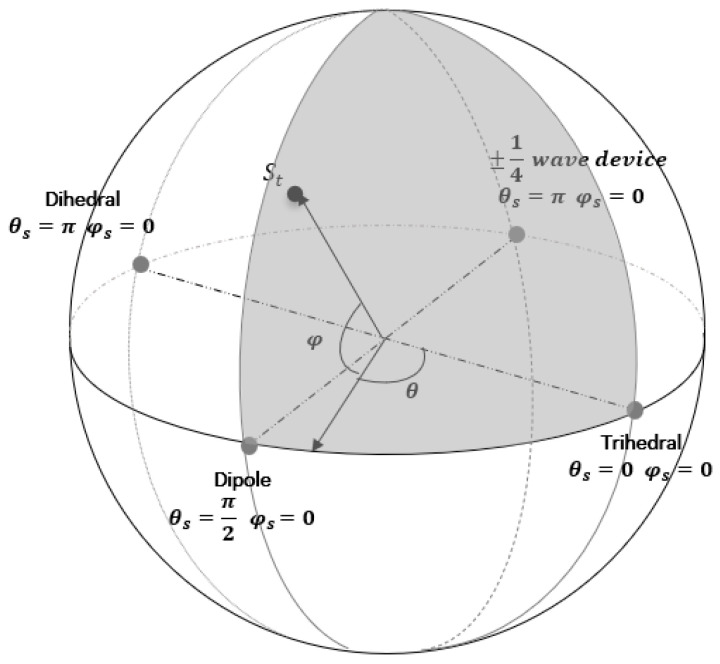
The novel spherical configuration constructed from the inherent complementarity among the elementary scattering mechanisms.

2. The process of mapping a point from the complex unit disk onto the surface of the unit sphere is elucidated here. The PolSAR cell being studied, along with its scattering matrix, is now represented by the longitude θ and the latitude φ on the unit sphere (Figure 12).

3. According to Poelman [73], the fundamental scattering characteristics of Cylinder and Narrow Diplane can be described as a linear combination of other elementary scattering mechanisms outlined in the Cameron classification scheme. Specifically, these scatterers encompass the trihedral, dihedral, and dipole:

(133) $$S_{cyl}\left(\varphi \right)=\frac{1}{2}S_{tri}+\frac{1}{2}S_{dip}\left(\varphi \right)$$

(134) $${\;S}_{ndi}(\varphi )=\frac{1}{2}S_{dih}(\varphi )+\frac{1}{2}S_{dip}(\varphi )$$

4. As the scattering mechanisms of Cylinder and Narrow Diplane can be composed of Trihedral, Dipole, and Dihedral, these three, along with the ¼ wave device, are considered fundamental scattering mechanisms. This assertion led us to dismiss the scattering mechanisms of the Cylinder and Narrow Diplane as having minimal significance and update the spherical topology as depicted in Figure 12. Based on the angle coordinates (θ,φ) of the point being analyzed, the identification of the right-angled spherical triangle it pertains to is established. Depending on its placement relative to the equator, one vertex of the triangle remains the pole of the sphere, while the other two vertices represent the closest scattering mechanisms determined using the orthodromic or great circle distance D:

(135) $$D=arccos\;(sin\varphi_{1}sin\varphi_{2}+cos\varphi_{1}\cos\varphi_{2}\cos\left(\Delta \theta \right))$$

5. The vector, originating from the center of the sphere and terminating at the coordinates on the spherical shell, is projected onto the equator level to which the reference scattering mechanisms belong, based on the angle φ (Figure 12). Specifically, the projection is confined within the quadrant delimited by the center of the sphere and the two nearest scatterers to the examination point.
6. An immediate outcome is the analysis of the vector’s projection into two vertical components, signifying the presence of the two nearest scattering mechanisms.

Based on the above procedure, the mixture interpretation for each scatterer is accomplished by:

(136) $$S_{t}=P_{1}S_{1}+P_{2}S_{2}$$

where $S_{1}$ and $S_{2}$ correspond to the primary and the secondary scattering mechanisms respective, while $P_{i}$, i=1,2 is calculated according to:

(137) $$P_{i}=cos\varphi_{i}\;cos\theta_{i}$$

It is worth mentioning that $P_{i}$ calculates the degree of contribution of each of the two dominating fundamental scattering mechanisms. As $P_{i}$ approaches 1 or 100% indicates that the target scatterer $S_{t}$ is completely characterized by one of the four fundamental scattering mechanisms. In the marginal case where $\varphi =90^{\circ }$, the scatterer can be assumed as undetermined and be classified as “non-Categorizable”.

The interpretation of the informational content within each PolSAR cell by a pair of two scattering mechanisms, as outlined in the Double Scatterer Model, is straightforward and dependable. Initially, both the novel method and the well-established Cameron’ technique were employed to generate color representations of the aforementioned data sets (Table 7 and Table 8). As evident from Table 7, every elementary scattering mechanism identified by Cameron and, consequently, each PolSAR cell is associated with a unique color. In contrast, the Double Scatterer Model employs only four symmetric elementary scatterers, interpreting each cell through a combination of the two most influential elementary scattering mechanisms and their weights/powers. This is illustrated by blending the colors corresponding to the four scatterers utilized in the present methodology, as outlined in Table 8, in a manner proportionate to the dominance of the scatterers within each cell. As anticipated, considering the complementary nature of the elementary scattering mechanisms within the spherical topology depicted in Figure 12, there are only eight potential scattering pairs involving primary and secondary mechanisms, detailed in Table 9.

The objective was to accentuate the finest details extracted through the proposed methodology. Consequently, a comprehensive analysis of the proposed method was conducted to emphasize the substantial information content that can be derived from the processing of full polarimetric data using the Double Scatterer Method.

**Figure 13:**
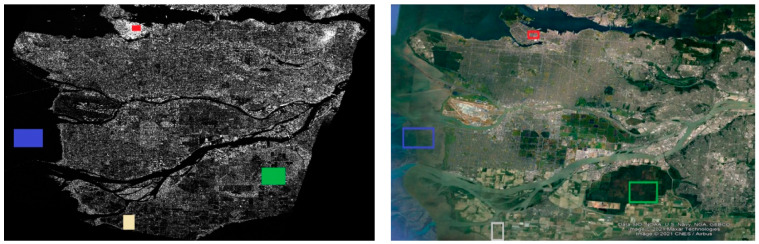
**Left**: Polarimetric geocoded SAR data covering the broader Vancouver region as it is depicted by the intensity of the HV channel. In this representation, water bodies are indicated by blue, forested areas by green, urban/built-up areas by red, and agriculture/pasture lands by beige. **Right** is the corresponding view on Google Earth.

Specifically, a partition of different land cover types based on the analytic description of the region of Vancouver obtained by the research of [74] was made. The selected region encompasses all the geological features specific to the area, serving as a general criterion to ensure the robustness and effectiveness of the proposed feature extraction process across various datasets. Consequently, four primary types of land cover were chosen for classification: water bodies, urban/built-up areas, forest/wooded areas, and agriculture/pasture areas. These regions are depicted in Figure 13.

The primary objective of this experimental procedure went beyond just analyzing visual differences. It aimed to explore the fundamental information provided by each method. As expected, based on Cameron’s deterministic approach, each polarimetric cell was assigned a numerical value between 1 and 8, corresponding to the specific scattering mechanisms he proposed. In homogenous areas like the sea, there was an expectation of similar scattering mechanisms across polarimetric cells. On the other hand, areas with more variations showed intriguing results.

In contrast, the Double Scatterer Model projected a more intricate picture. Each polarimetric cell was interpreted by a composite of two scattering mechanisms along with their corresponding weights. This technique yielded a set of four numerical values for each PolSAR cell. The ultimate objective was to ensure that this mode of interpretation showcased substantial discriminability, ideally culminating in a unique characterization for each land cover type.

**Figure 14:**
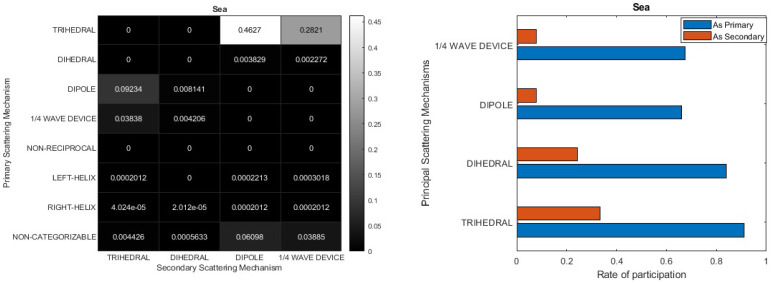
The participation rate of each Primary–Secondary scattering pair on the **left** side (Heatmap representation) and on the **right** side (bar diagram representation) for each scattering mechanism (pixel) in the sea.

Figure 14 provides information on the primary and secondary scattering mechanisms for sea. The heatmap in the top-left corner of the figure clearly shows that the Trihedral is the primary scattering mechanism in approximately 74% of cases. This percentage is obtained by adding the instances where the trihedral is identified as the primary scattering mechanism, such as in the scattering pairs trihedral-dipole 0.4627 and trihedral-1/4 wave device 0.2821. Simultaneously, the Trihedral appears as a secondary scattering mechanism for another ~13%. The Trihedral is the main scattering mechanism in the large majority of the PolSAR cells in the sea, with the Dipole and the ¼ Wave Device having a small participation. The criterion of non-reciprocity holds true for all the polarimetric cells under examination. Simultaneously, the count of non-symmetric scatterers—namely, the left and right helices—each of which does not form pairs with other scattering mechanisms, remains notably low. Similarly, the number of PolSAR cells that resist categorization is minimal. These findings were as anticipated, particularly when studying the expanse of the sea, which showcases remarkable homogeneity and minimal deviations. Additionally, in Figure 14 in the bar diagram at the upper right is given information regarding the average strength of each scattering mechanism. This encompasses cases where a mechanism is identified as the primary scatterer as well as instances where it functions as a secondary scatterer within each PolSAR cell. According to this bar diagram, the Trihedral in the cells in which it is the dominating scattering mechanism has a participation as it is expressed by the coefficient $P_{i}$ in Equations (136) and (137), exceeding 90%. As a secondary scattering mechanism the coefficient $P_{i}$ is around 30% or 0.3. The dihedral as primary scatterer has the second highest contribution, while its role as secondary scatterer reaches 25%, underscoring the importance of its scattering behavior. In contrast, the Dipole and 1/4 wave device show notably lower percentages. Certainly, the double scatterer representation offers richer and more detailed information compared to Cameron’s representation.

**Figure 15:**
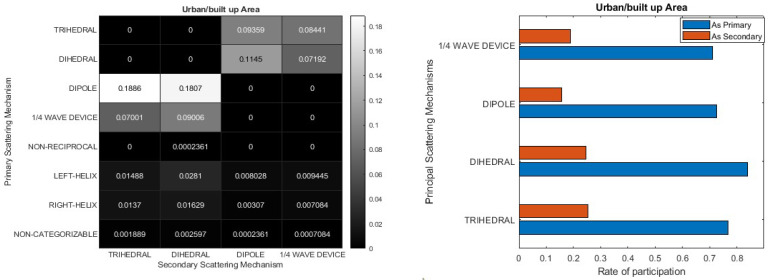
The participation rate of each Primary-Secondary scattering pair in the **left** side (Heatmap representation) and on the **right** side (bar diagram representation) for each scattering mechanism (pixel) in an urban/built-up area.

In a similar manner, Figure 15 illustrates the information concerning primary and secondary elemental scatterers for the urban area. According to the depiction, the Dipole emerges as the primary scattering mechanism, constituting approximately 36% of the total, with the Trihedral and Dihedral serving as secondary scattering mechanisms. Conversely, the last two mechanisms appear in reverse order as primary scattering mechanisms, accounting for approximately 17% probability in total, and their secondary scattering mechanisms include the Dipole and the ¼ wave device. Notably, over 20% of the PolSAR pixels either cannot be confidently classified into any elementary scattering mechanism or are identified as non-symmetric (left/right helix), or do not adhere to the reciprocity criterion. In the context of the urban/built-up scenario, the significance of the second scattering mechanism becomes evident in extracting valuable information. The proposed double scatterer method introduces a spectrum of nuances within each cell, exhibiting great promise in intricate settings like urban areas.

**Figure 16:**
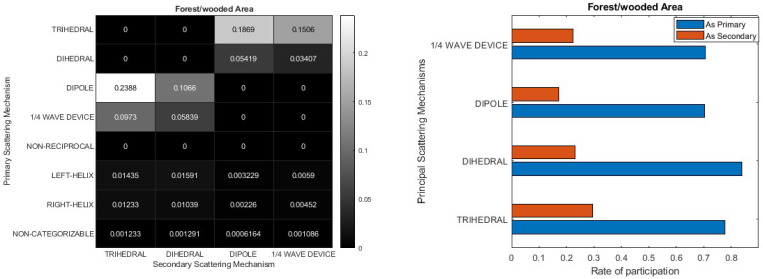
The participation rate of each Primary–Secondary scattering pair on the **left** side (Heatmap representation) and on the **right** side (bar diagram representation) for each scattering mechanism (pixel) in a forest/wooded area.

Figure 16 presents information concerning primary and secondary scattering mechanisms in forested areas. The heatmap on the left side of Figure 16 reveals that the Dipole and Trihedral serve as primary scattering mechanisms, accounting for approximately 34% of the total. Among the other elementary scatterers, the ¼ wave device assumes a primary role with an occurrence rate of about 14%. Meanwhile, the Dihedral appears less frequently as a primary mechanism compared to others, yet the bar diagram indicates its more substantial contribution. Notably, the second scattering mechanism, regardless of its identity, participates at a level of approximately 20%, except for the Trihedral, which exhibits a more prominent role as a secondary scatterer (around 30%). As observed in the urban area, a similar situation is evident in this region, where homogeneity is notably absent. Approximately 20% of the PolSAR pixels exhibit characteristics that make them challenging to confidently classify into any elementary scattering mechanism. Alternatively, these pixels are identified as non-symmetric (left/right helix) or fail to adhere to the reciprocity criterion.

**Figure 17:**
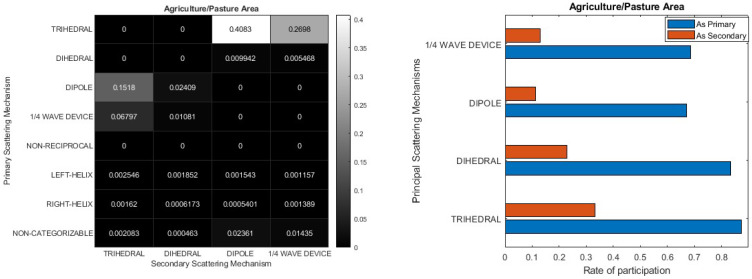
The participation rate of each Primary-Secondary scattering pair on the **left** side (Heatmap representation) and on the **right** side (bar diagram representation) for each scattering mechanism (pixel) in a forest/wooded area.

Finally, Figure 17 provides insight into the primary and secondary scattering mechanisms within the context of agricultural land cover. Given the flat nature of this land cover type, a certain resemblance to the sea, as depicted in Figure 14, is to be anticipated. This parallel is evident upon comparing the two heatmaps (Figure 14 and Figure 17), where the Trihedral mechanism stands out dominantly, yet with a heightened contribution and a greater presence of non-categorizable and asymmetric scatterers in the agriculture/pasture area. Moreover, in marine environments, the overwhelming predominance of the Trihedral mechanism is evident from the sheer clarity of the blue color. Conversely, in agricultural regions, the influence of the second most prominent scattering mechanism is noticeable, as it is reflected in several darker shades.

The Double Scatterer Model, as a recently introduced tool for fully polarimetric data processing, has demonstrated its efficacy in two distinct applications. Specifically, it has proven highly successful in land cover classification, as evidenced by [75], where the polarimetric information extracted using the Double Scatterer Model was employed in a simple neural network, resulting in exceptional accuracy. Additionally, Karachristos and Anastassopoulos [76] explored the efficiency of this novel algorithm in a target detection framework, yielding remarkable results. These instances underscore the robustness of the Double Scatterer Model, establishing it as a highly promising method for polarimetric data processing, excelling in both classification and detection tasks. Exploring the extension of the Double Scatterer Model to novel applications, such as urban monitoring or environmental assessment, may uncover new insights into its versatility. Future work could also focus on validating and refining the model through comprehensive experiments and real-world case studies, ensuring its reliability and applicability in diverse remote sensing scenarios. Overall, the ongoing evolution of the Double Scatterer Model is crucial for pushing the boundaries of PolSAR data analysis and interpretation. A concise summary of its advantages, disadvantages, and application capabilities is presented in Table 10.

## 7. Conclusions

In recent decades, numerous polarimetric decomposition algorithms have been suggested, indicating the increasing interest in extracting valuable information from satellite data. The continuous advancements in technology employed during the data acquisition process have greatly enhanced the informative content of satellite data, leading to the development of various information extraction methods. This study focuses on analyzing five well-established techniques and introducing a new approach called the Double Scatterer Model. Each algorithm offers unique advantages but also exhibits certain drawbacks. The Pauli decomposition, for instance, is a simple approach that provides a solid physical interpretation. However, its limitations in interpreting asymmetric scattering behavior and utilizing a limited number of scattering mechanisms make it more suitable for optical presentations rather than tasks that require high accuracy levels. On the other hand, the Cameron decomposition allows for a greater utilization of elemental scattering mechanisms, but in real-world scenarios, many objects do not exclusively correspond to these elementary scattering mechanisms. Additionally, ambiguities related to mathematical topology and the metric used in Cameron decomposition discourage relying solely on this technique for experiments. Moreover, it should be noted that all coherent decomposition methods are susceptible to noise, which cannot be effectively addressed by first-order statistics. Regarding the non-coherent approach, as mentioned by authors Freeman and Durden, the three-component model demonstrates better results in extracting terrain information. However, its validity is highly dependent on several restrictions. Yamaguchi’s approach proves to be more efficient as an “extension” of the Freeman and Durden method. Nonetheless, its applicability is limited by the potential inconsistency of the components it relies on. The H/a decomposition emerges as a highly efficient approach due to its strong mathematical foundation and the utilization of stochastic analysis in matrix decomposition. Nevertheless, it is based on several assumptions, such as the assumption of Bernoulli parallelism, which raises questions as to whether such mathematical processes are strictly governed by well-defined concepts. However, in both model-based and eigenvector analysis approaches, it is worth noting that the utilization of second-order statistics allows for various speckle denoising procedures, thereby increasing the validity of the procedures. As for the Double Scatterer Model, it is an approach that aims to combine the deterministic nature of coherent decomposition with the robustness of non-coherent methods. Initial results appear promising, but further in-depth analysis is required to fully assess its effectiveness. In summary, the initial hypothesis has been substantiated. The approach of interpreting each PolSAR cell through a blend of the two most influential scattering mechanisms has proven to be highly satisfactory, enabling a more comprehensive analysis. This method harnesses a wealth of information, allowing for a deeper exploration of the scattering characteristics exhibited by each area or target. The conclusions drawn from the utilization of the Double Scatterer Model to represent the information contained in each PolSAR are robustly affirmed through the coloring that has been employed. By examining the contribution of the two dominant scatterers in each pixel/cell, corresponding to specific colors, a nuanced representation is achieved. This detailed depiction, capturing all possible combinations of colors (as delineated in the 8 combinations in Table 9), underscores the pivotal role of the proposed tool/feature in both the classification and target detection processes. This is attributed to the tool’s ability to provide a comprehensive and detailed analysis, enhancing our understanding of the data.

In today’s era, the prevalence of machine learning algorithms has brought forth new approaches that combine information extraction with high-accuracy algorithms. This emerging trend, coupled with the availability of high-quality data, has proven to be the most efficient procedure. An ideal framework would encompass multiple data sources, employ a variety of information extraction approaches, and leverage the sophisticated nature of machine learning.

## Figures and Tables

**Table 1:** Elementary scattering mechanisms according to Cameron Decomposition.

Elementary Scatterer	Normalized Complex Vector	Complex Parameter z
Trihedral	$\hat{\Lambda }\left(1\right)$	1
Dihedral	$\hat{\Lambda }(-1)$	−1
Dipole	$\hat{\Lambda }(0)$	0
Cylinder	$\hat{\Lambda }(+\frac{1}{2})$	$+\frac{1}{2}$
Narrow Diplane	$\hat{\Lambda }(-\frac{1}{2})$	$-\frac{1}{2}$
¼ wave devise	$\hat{\Lambda }(\pm j)$	±j

**Table 2:** The elementary scattering mechanisms outlined in Cameron’s CTD, and the corresponding color scheme employed to represent them.

Scattering Mechanisms by Cameron	Color Representation
Trihedral	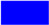
Dihedral	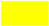
Dipole	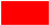
Cylinder	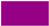
Narrow Diplane	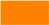
¼ wave device	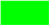
Left helix	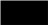
Right Helix	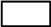

**Table 3:** Summarization of Coherent Target Decompositions.

Coherent Target Decomposition		**Advantages**	**Disadvantages**	**Application Fields**
	Pauli Decomposition	Can effectively differentiate natural targets	Unable to identify artificial targets	Image coloring
		Dependency on the orientation angle	Not all scattering behaviors can be explained	Easily combine with machine learning algorithms
		Computationally straightforward		
	Cameron Coherent Target Decomposition	Optimize the utilization of data from the maximized symmetric component of coherent targets	Not all scattering behaviors can be explained	Ship detection
		Additional scattering mechanisms for interpreting scattering behaviors	Greater computational cost than Pauli	Easily combine with machine learning algorithms
			Not appropriate for intricate situations involving asymmetric targets	

**Table 4:** The covariance matrix of the volume scattering as it is determined based on the ratio $10log(\frac{\langle {\left|S_{VV}\right|}^{2}\rangle }{\langle {\left|S_{HH}\right|}^{2}\rangle })$.

$10\log(\frac{\langle {\left|S_{VV}\right|}^{2}\rangle }{\langle {\left|S_{HH}\right|}^{2}\rangle })$	−4 dB	−2 dB	−2 dB	2 dB	2 dB	4 dB
${\left[C\right]}_{vol}$	$\frac{1}{15}\left[\begin{array}{ccc} 8 & 0 & 2\\ 0 & 4 & 0\\ 2 & 0 & 3 \end{array}\right]$		$\frac{1}{8}\left[\begin{array}{ccc} 3 & 0 & 1\\ 0 & 2 & 0\\ 1 & 0 & 3 \end{array}\right]$		$\frac{1}{15}\left[\begin{array}{ccc} 3 & 0 & 2\\ 0 & 4 & 0\\ 2 & 0 & 8 \end{array}\right]$	

**Table 5:** Summarization of Non-Coherent Model-Based Target Decompositions.

Non-Coherent Target Decomposition Model-Based Approaches		**Advantages**	**Disadvantages**	**Application Fields**
	Freeman–Durden Decomposition (Three component Model)	Based on fundamental principles of radar scattering	Unable to distinguish forest and man-made buildings	Land use–land cover Forest and crop monitoring
		Distinguish various surface cover types	The validity of the three components it relies upon may not always hold	
		Suitable for natural distributed target areas analysis	The accuracy of the results depends on the correlation coefficients, which assume reflection symmetry	
	Yamaguchi Decomposition (Four component Model)	Extended Three Components Decomposition	Sensitivity to noise	Natural disaster monitoring
		Additional scattering mechanisms	Greater computational cost	Terrain Classification
			Dependence on specific assumptions which may not hold in all situations.	

**Table 6:** Summarization of Cloude–Pottier Entropy Based Decomposition.

Non-Coherent Target Decomposition Eigenvector-Eigenvalue Approaches		**Advantages**	**Disadvantages**	**Application Fields**
	Cloude–Pottier Entropy-based decomposition	Detailed information about scattering mechanisms	Complex mathematical formulations	Land cover classification
		Physical interpretations	Dependence on Assumptions	Environmental Monitoring
		Limited Sensitivity to Certain Targets	Limited Sensitivity to Certain Targets	Target Recognition

**Table 7:** Assigning colors to the elementary scattering mechanisms specifically proposed by Cameron in CTD.

Scattering Mechanism	Color Representation
Trihedral	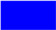
Dihedral	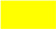
Dipole	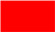
Cylinder	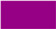
Narrow Diplane	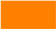
¼ wave device	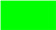
Left helix/Right helix	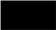

**Table 8:** Color mapping corresponding to the elementary scatterers employed in the Double Scatterer method.

Proposed Scattering Mechanism	Color Representation
Trihedral	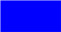
Dihedral	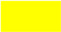
Dipole	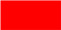
¼ wave device	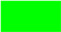
Left helix/Right helix	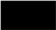

**Table 9:** Mapping colors to the PolSAR cells that are interpreted as a combination of the primary and secondary scattering mechanisms.

Primary Scattering Mechanism	Secondary Scattering Mechanism	Color Representation
Trihedral	Dipole	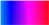
Dipole	Trihedral	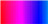
Trihedral	¼ wave device	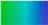
¼ wave device	Trihedral	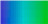
Dihedral	Dipole	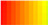
Dipole	Dihedral	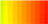
Dihedral	¼ wave device	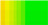
¼ wave device	Dihedral	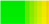
Asymmetric Scattering Mechanisms		
Left helix		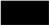
Right helix		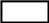

**Table 10:** Summarization of Double Scatterer Model Decomposition.

Double Scatterer Model	**Advantages**	**Disadvantages**	**Application Fields**
	Deep analysis of the scattering nature of each PolSAR cell	Noise sensitivity	Land cover classification
	Stepwise procedure	Boundless potential for evaluation due to its innovative nature	Image Segmentation
	Efficient and versatile feature extraction method		
	Versatile in its applicability	Limited Sensitivity to Certain Targets	Target Recognition

## Data Availability

Data availability upon request.
